# Application of value stream mapping to design and develop an inventory management system in a hospital

**DOI:** 10.12688/f1000research.159736.1

**Published:** 2025-03-31

**Authors:** Praewchit Ritthaisong, Nusaraporn Kessomboon

**Affiliations:** 1 Khon Kaen University, Nai Mueang, Khon Kaen, Thailand

**Keywords:** Value Stream Mapping (VSM), Lean Management, Inventory Management System, Medical supply warehouse.

## Abstract

**Background:**

Insufficient drug reserves in hospitals ready for immediate use pose significant challenges in the management, procurement, and distribution of medications. Therefore, there is a critical need for improved resource management processes, ensuring a balanced and efficient medication reserve system. Lean Management is a systematic approach designed to eliminate waste, thereby reducing costs and enhancing efficiency, making it more effective than other management systems. Value Stream Mapping (VSM) is a crucial tool within the Lean system, specifically designed to support the development of manufacturing strategies. This approach aids in identifying and categorizing activities as value-added (VA), necessary but non-value-added (NNVA), or non-value-added (NVA). In this study, VSM was employed to comprehensively analyze the drug disbursement process within the medical supply warehouse managementsystem.

**Method:**

This research conducted using action research methodology. This phase primarily focused on the initial step of planning, outlining the conceptual framework for the research. Regarding potential improvements, it is expected that enhancing the satisfaction period concerning the waiting time for those involved will likely increase sufficiency and availability, making work more convenient.

**Result:**

Phukhieo Chaleomphrakeit Hospital was able to draw a map of the value stream in the future state, adjusting from 7 steps to 6 steps. The total time in the current system is 1,925 minutes. However, when designing and planning the future state of the pharmaceutical inventory management system, it is expected that the total time spent in the system will be reduced to only 435 minutes, with time spent on valuable activities reduced to 395 minutes. The percentage of time spent on valuable activities is 20.52%, which reduces waste in waiting.

**Conclusion:**

Design of work systems by those involved. This is a tool that helps reduce waste and has been developed collaboratively to make operations run more smoothly.

## Introduction

Insufficient drug reserves in hospitals ready for immediate use pose significant challenges to the management, procurement, and distribution of medications, particularly those required for chronic diseases. The increasing demand for specific medications further complicates this issue, often leading to inadequate reserves and potential shortages. In response, hospitals may overcompensate for stockpiling medications, resulting in excessive inventory and medication expiration. Therefore, there is a critical need for improved resource management processes that not only mitigate shortages but also anticipate and prevent overstocking, ensuring a balanced and efficient medication reserve system.

Phukhieo Chaleomphrakeit Hospital, located in Chaiyaphum Province, is a small general hospital (M1) with 300 beds that, serves the entire Phukhieo District. It also acts as a host hospital, overseeing a network that includes Khon San Hospital, Kasetsomboon Hospital, and Ban Tan Hospital, along with 15 subdistrict health promotion hospitals and one primary care unit. Recognized as a model hospital for its commitment to quality, safety, and positive patient feedback, it operates with seven strategic objectives, including a focus on providing cost-effective services. As of October 2021, the hospital has managed a list of 588 items. There are two service units with sub-stock: outpatient and inpatient medicine rooms. Medicines are delivered to these sub-depots once a week, leading to large reserves and a lack of clear inspection protocols. Consequently, there have been instances of drug shortages despite the availability of drugs in warehouses, with approximately one emergency reported per week outside the delivery schedule. In 2021 alone, 59 drug emergencies involving 164 items for the Outpatient Medicine Room and 66 emergencies involving 133 items for the Inpatient Medicine Room were reported. Moreover, 28 medicine items were unavailable, contributing to an increasing number of expired medicines in the warehouse, totaling 51 items per year. Managing unknown quantities and shelf-life of drugs pose challenges, exacerbated by an annual increase in drug inventory value. By the end of the fourth quarter of 2021, the drug reserve rate had risen to 2.46 months, up from 1.66 months in the same period of 2020, highlighting excess reserves exceeding the optimal limit of two months. The medical disbursement process within the hospital has been identified as lengthy and complex, contributing to insufficient readiness for use and placing a disproportionate workload on available personnel. Analysis of current workflows has identified inefficiencies, prompting the development of a plan to implement new systems aimed at reducing waste and enhancing the efficiency of drug distribution.

Lean Management is a systematic approach designed to eliminate waste, thereby reducing costs and enhancing efficiency, making it more effective than other management systems ( Lanza-León, P., et al., 2021) delineate this system based on five core principles. The first principle, Specify Value, defines or add value from the service recipient’s perspective. The second principle, Identifying the Value Stream, focuses on analyzing and identifying the value stream to detect, minimize, or eliminate waste. The third principle, flow, aims to optimize the work processes for continuous and uninterrupted flows. The fourth principle, pull, emphasizes initiating processes based on the demand or readiness of the subsequent step rather than pushing tasks forward. Finally, the fifth principle, Pursue Perfection, encourages striving for continuous improvement and applying these principles flexibly in any organizational context, whether in production or services. Lean Thinking seeks to create value by eliminating waste and enhancing organizational flexibility across the entire process. This underscores the ongoing reduction of waste and the continual improvement of each critical structure, following a cyclical approach ( Womack, J. P., & Jones, D. T., 1996; Womack, J. P., & Jones, D. T., 2003). Since its application in the healthcare sector in 2009, Lean Management has demonstrated notable benefits, including reduced waiting times, decreased unnecessary hospital admissions, lower costs, and fewer errors. Moreover, thier adoption has bolstered the quality, safety, and efficiency of clinical processes ( Lanza-León, P., et al., 2021)

Value Stream Mapping (VSM) is a crucial tool within a lean system, specifically designed to support the development of manufacturing strategies. Unlike other process maps, VSM creates visual representations that analyze the flow of both raw materials and information, providing a comprehensive overview of the entire process flow. It focuses on illustrating how activities within the system deliver value to customers, emphasizing the flow of resources and information across the supply chain. Value Stream Mapping enables a holistic understanding of processes, highlighting both the duration and proportion of time allocated to each activity. This approach aids in identifying and categorizing activities as value-added (VA), necessary but non-value-added (NNVA), or non-value-added (NVA) ( Martin, K., & Osterling, M., 2014). By pinpointing non-value-added activities as an initial step, VSM facilitates the identification of waste within production systems ( Shararah, M. A., 2013). Value Stream Mapping (VSM) serves as a guideline for classifying activities that enhance value creation and minimize waste in critical processes. It involves analyzing the current state of processes and documenting improvements to define a future state. This process analysis employs collaborative teamwork principles to gain insights from the customer's perspective and optimize the flow of resources and information throughout the supply chain.

Value Stream Mapping (VSM) is a lean management approach that enhances patient values and care quality when integrated into healthcare processes. In public health settings, VSM aims to eliminate waste by minimizing wait times, avoiding unnecessary steps, and adding value this is directly beneficial to patients. By reducing the non-value-added time, VSM significantly improves process quality and care outcomes, focusing on patient-centered care to enhance satisfaction, particularly by reducing long wait times ( Nowak, M., et al., 2017).

Before implementing VSM, it is crucial to educate and train employees on lean concepts and objectives to alleviate concerns and resistance, thereby positively influencing organizational processes and quality outcomes in healthcare settings ( Gellad, Z. F., & Day, T. E., 2016). Furthermore, VSM helps improve manufacturing efficiency at the supplier endpoint by reducing production manpower, cutting operation time, minimizing waiting time, and promoting better operational practices, leading to reduced manufacturing costs ( Seth, D., & Gupta, V., 2005). This approach has proven effective across various industries beyond healthcare, including textiles, pharmaceuticals, food, telecommunications, and oil and gas, demonstrating its versatility in optimizing process metrics such as inventory levels, lead times, and overall efficiency ( Batwara, A., et al., 2023).

In healthcare, particularly in hospitals and clinics, VSM serves as a vital tool for visualizing workflows and identifying inefficient processes. It is instrumental in mapping out current states and recommending improvements that streamline operations and enhance patient care delivery ( Gill, P. S., 2012). The process of creating a VSM involves multidisciplinary collaboration to ensure comprehensive identification of non-value-added activities and foster a culture of continuous improvement among staff, aligning with lean principles of empowering workers to lead change efforts ( Gellad, Z. F., & Day, T. E., 2016).

Hospitals worldwide are increasingly emphasizing the application of lean concepts in healthcare management. Research has been conducted in hospitals in Brazil using Value Stream Mapping (VSM) to analyze healthcare environments. The results of this study indicate that the proposed VSM model can identify bottlenecks in operations and various forms of waste, leading to insights into operational inefficiencies and bottlenecks ( Henrique, D.B., et al., 2016).

A study was conducted in Rajasthan, India, to implement Supply Chain Value Stream Mapping (SCVSM) for government-sponsored drugs in the Drug Distribution System (DDS). Data were gathered through direct observations, document analysis, and semi-structured interviews. The SCVSM model developed aimed to optimize lean practices and gain government support for healthcare supply chain efficiency in India. This mixed-methods study identified non-value-added activities in the value stream and demonstrated the effectiveness of SCVSM in monitoring waste to ensure timely and high-quality drug delivery.

The study concluded that implementing Kaizen methodologies improved the SCVSM by addressing waiting times and other inefficiencies. Four phases of research have categorized DDS activities into Process, Transport, Delay, Inspection, and Storage, revealing opportunities for improvement. Following the VSM guidelines, future SCVSM activities were reduced from 27 to 16 after the Kaizen implementation, leading to a 7.14% reduction in the total product lead time. The Identified non-value-added activities included waiting periods in transportation, improper processes, unnecessary inventory, and movements. the proposed future SCVSM maps aim to eliminate these inefficiencies, potentially reducing the drug delivery times and operational costs ( Dixit, A., et al., 2021).

A study conducted at a state-of-the-art government pharmaceutical organization in Malaysia focused on optimizing pharmaceutical warehousing operations using value stream mapping (VSM). This study aims to explore the feasibility of implementing lean practices to enhance efficiency and eliminate waste in warehouse operations. VSM, known for its effectiveness in identifying value-adding activities and improving process flow, facilitates the mapping of operations and data collection through interviews and observations. The study revealed significant improvements: the total processing time decreased from 45,420 to 11,940 min, and the non-value-added time was reduced from 21,060 to 3,120 min. Workforce requirements also decreased from 51 to 31 (39.21%). The implementation of Kaizen activities, along with effective policies and decisions, further contributed to these improvements. This case study demonstrates VSM's utility of VSM in identifying and eliminating wasteful steps, such as unnecessary inventory, machinery, storage space, and excessive processes, thereby fostering continuous improvement in warehouse operations within the pharmaceutical supply chain context ( Abideen, A. Z., & Mohamad, F. B., 2019). A study conducted at a university in Berlin, Germany, applied Value Stream Mapping (VSM) to reduce waste in the procurement process of tube expander coils (endovascular stents). The current state analysis using the VSM identified 13 steps in the procurement process. Among these, only two steps were recognized as value-adding activities, where as five steps were identified as non-value-adding activities ( Teichgräber, U. K., & de Bucourt, M., 2012).

VSM has been studied to assess potential improvements in the drug coordination process with the aim of reducing non-value-added actions and optimizing medication synchronization across multiple independent community pharmacies. This study utilized an observational cross-sectional design employing VSM to provide a detailed depiction of each step of the medication coordination process. This research involved observing the time required for processing, packaging, and inspecting prescription drugs on different days and at various times, both before and after the interventions were implemented. As a result of these interventions, the two process steps were eliminated, leading to reduced packaging times. This demonstrates that Value Stream Mapping is a valuable tool for identifying and eliminating non-value-adding activities, thereby improving operational efficiency and standardization ( Renfro, C.P., et al., 2022)

In terms of publications, VSM has been extensively studied and applied. A review of studies conducted between 2015 and 2019 revealed that the majority of VSM applications are documented in tertiary-level contexts. The United States has emerged as the primary country where these applications are reported, with variations observed in VSM development across different geographical regions. Sustainability indicators predominantly encompass operational and social aspects, highlighting the gap in the inclusion of environmental indicators. There is a pressing need for greater standardization in the application of VSM within the healthcare sector, particularly for integrating environmental metrics ( Batwara, A., et al., 2023).

In Thailand, several studies have explored the application of VSM in healthcare service procedures, focusing specifically on improving hospital operations and outpatient drug dispensing services. For instance, a study conducted at Nopparat Ratchathani Hospital examined the efficiency of using VSM to analyze waste and add value to drug dispensing services. Before improvement, the average waiting time for receiving medicine was 54.01 ± 11.24 minutes. Following improvements facilitated by VSM, this waiting time decreased significantly to 46.11 ± 24.45 minutes, demonstrating a statistically significant improvement ( Chanbancherd, P., et al., 2012).

Similarly, at Borabue Hospital in Mahasarakham, VSM was use to develop the drug disbursement process with the aim of identifying and reducing waste. Analysis of the ten work steps revealed that only three activities were value-adding, four activities were non-value-adding, and three were necessary but did not add value. Value-adding activities accounted for 30.60% of the total lead time, prompting the redesign of the work system to enhance efficiency ( Jitthananan, K., 2017; Jitthananan, K., et al., 2017).

This study employed VSM to comprehensively analyze the drug disbursement process within the inventory management system, and identify existing waste and planning strategies for waste reduction. The aim is to enhance the efficiency of the medical supply inventory management system.

The objective of this study was to identify inefficiencies and challenges within the medical supply inventory management system, to reduce waste and optimize processes using Value Stream Mapping (VSM), and to design and develop an enhanced drug inventory management system to improve efficiency and effectiveness.

## Method

This research was approved by Center for Ethics the Human Reserch of Khon Kaen University on January 27, 2023, referene no. is HE652237 and was conducted using action research methodology based on the concepts proposed by Kemmis and McTaggart (1988) ( Kemmis, S.,et al., 2014). Action research is characterized by its participatory approach, which aims to enhance the efficiency and effectiveness of operational practices by analyzing current conditions and addressing existing challenges ( Chatakarn, V., 2015). It involves active engagement in problem-solving within real-world contexts, fostering continuous improvement in operational quality ( Johnson, AP, 2012). Action research typically involves four iterative steps: Planning, Action, Observation, and Reflection. These steps form a cyclical process of self-reflection and iterative improvement, in which each cycle informs subsequent actions and refinements ( Kemmis, S., & McTaggart, R., 1988). In this study, Value Stream Mapping (VSM) ( Martin, K., & Osterling, M., 2014) was employed to comprehensively analyze the drug disbursement process within the medical supply warehouse management system. This phase primarily focused on the initial planning step, outlining the conceptual framework of the research.

1. Engage in discussions and collaborative planning with pharmaceutical warehouse staff to understand their challenges. Consult with key personnel from the drug warehouse, outpatient medicine room, and inpatient medicine room, involving 22 individuals. The researchers provided an explanation of the study and obtained informed consent from all participants involved in the discussion before conducting the study, using a written consent form. All participants were required to sign the consent form, which was approved by the ethics committee. Gather insights into their operational needs and areas requiring improvement in medical warehouse management.
2. Plan the development of a user-centric system through focus group discussions aimed at identifying and prioritizing improvement opportunities. Conduct joint cause analysis sessions to effectively address the identified issues in routine operations.
3. The current inventory management process was studied and analyzed comprehensively using Value Stream Mapping (VSM). The existing workflow across all stages was mapped to pinpoint inefficiencies and areas of improvement in drug supply warehouse operations.
4. Integrate findings from the VSM analysis into the design of a future state value stream map for an enhanced inventory management system. Propose adjustments, such as transitioning from weekly to daily medication delivery to outpatient and inpatient medicine rooms. This change aims to minimize inventory levels in the dispensary and enhance the traceability of drug reserves. Streamline processes between the drug supply warehouse and pharmaceutical assistants by eliminating manual surveys and requisition writing.
5. Implement VSM as per the methodology outlined by Martin and Osterling (2014), incorporating five essential steps to systematically identify and mitigate waste in the inventory management system.

The VSM tool involves five steps, as outlined by Martin and Osterling (2014).

Step 1: Setting the Stage and Enabling Success

• A team comprising six drug warehouse employees and 22 participants was involved in setting goals and understanding the existing inventory management system.
• Officials from the medical supply warehouse section, pharmacists, pharmaceutical officers, and staff from the outpatient and inpatient medicine rooms were included.
• Analyze operational problems by explaining workflows in each responsible section and plan initial data collection through observations.
  Step 2: Understanding the Current State
• Engage all 22 officials in surveying and observing operations, with drug warehouse staff focusing on prescription receipt for medicine delivery to outpatient and inpatient rooms.
• Record the observed operations and document the flow of activities from the drug warehouse to the substock of the medicine rooms.
• Study and analyze the process diagram of the current inventory management system across all steps, identifying operational issues and gathering improvement needs.
• Conduct focus group discussions to jointly analyze causes and propose solutions for routine work issues.
• Create a value stream map using Visio 2013 to visualize the workflow from the medical supply warehouse to the two medicine room sub-stocks.
• Discuss and address questions regarding process issues, identify waste steps, and establish timelines for each activity.
  Step 3: Designing the Future State
• Develop an efficient work system by eliminating unnecessary steps, simplifying processes, and utilizing information management to reduce personnel involvement in certain steps.
  Step 4: Developing the Transformation Plan
  Step 5: Achieving and Sustaining Transformation
  In this study, steps 1-3 were utilized according to Martin and Osterling (2014) to design a future work system that enhances efficiency.

## Results

**Figure 1.**
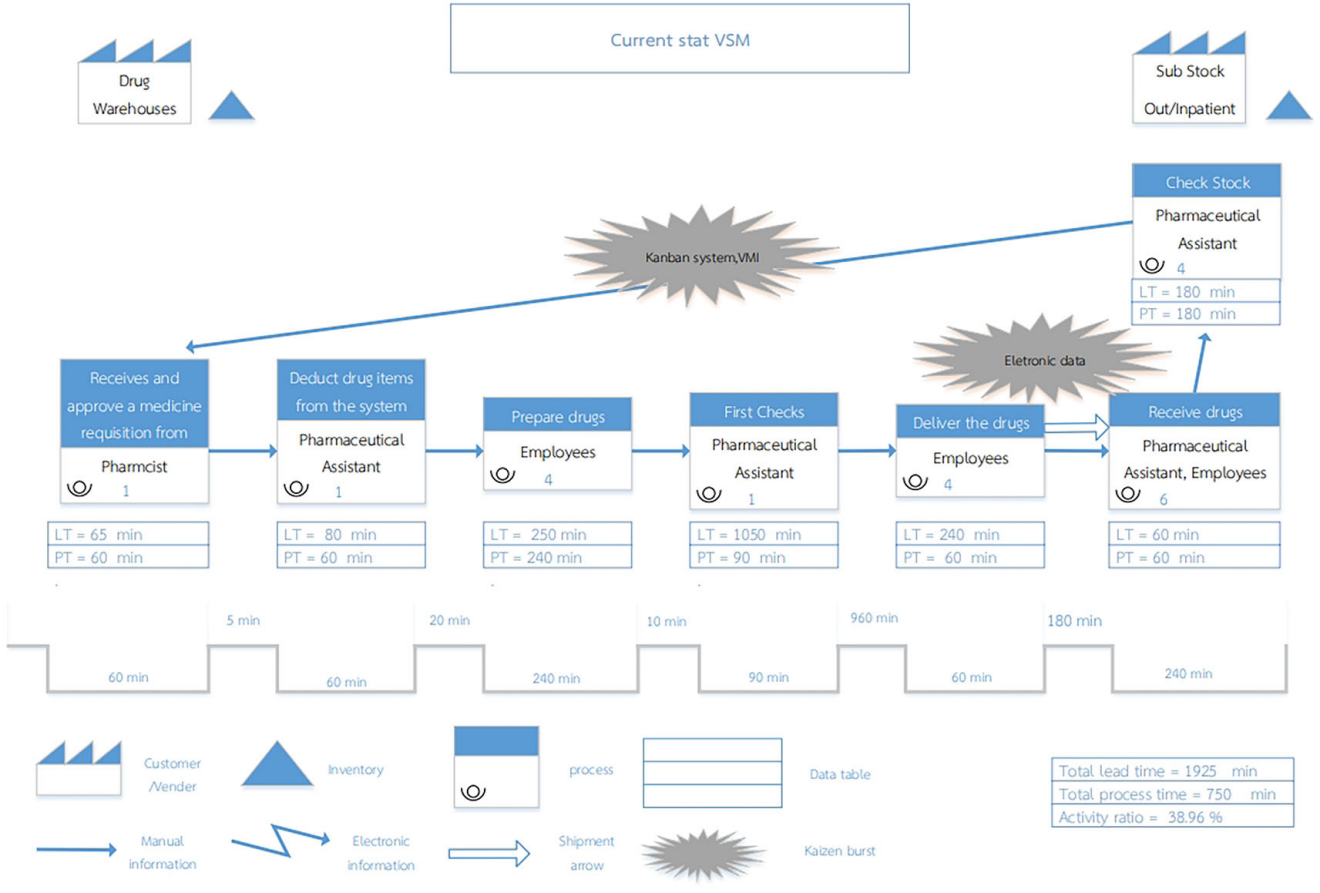
Current State Value Stream Mapping of the Inventory Management System at Phukhieo Chaleomphrakeit Hospital.


During the focus group discussions involving twenty-two staff members, several critical issues with the current medical supply inventory management system were identified. The primary concerns included the practice of stocking medicines weekly, which led to maintaining a high inventory value. This approach often results in shortages of essential medicines, necessitating emergency restocking between regular supply cycles. Furthermore, staffing shortages exacerbate the workload, particularly during peak times when large quantities of drugs are dispensed. The inefficiencies caused by these practices were evident in the significant amount of manpower and time required to store drugs in the warehouse. Moreover, the weekly disbursement schedule created spikes in workload, increasing the risk of medication errors and straining the available storage space. These findings underscore the need to redesign the inventory management system to effectively address these operational challenges ( Figure 1).

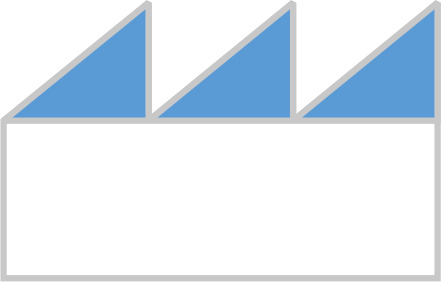
     = The Customer/Supplier relationship defines the drug warehouse as the supplier currently serving as the service provider for dispensing drugs to customers, namely the two medicine rooms.


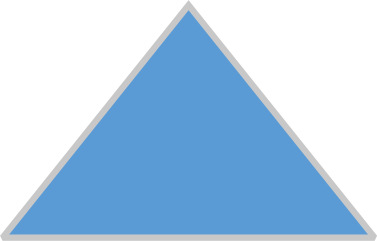
      = Inventory, refers to medicines held in reserve, encompassing drug warehouses, outpatient medicine rooms, and inpatient medicine rooms.


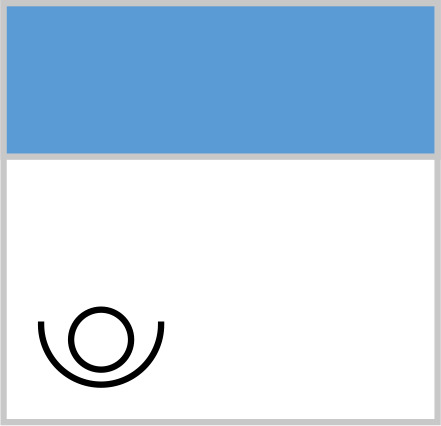
     = In the current state, the process depicts each step in the workflow of the system, which comprises seven steps.


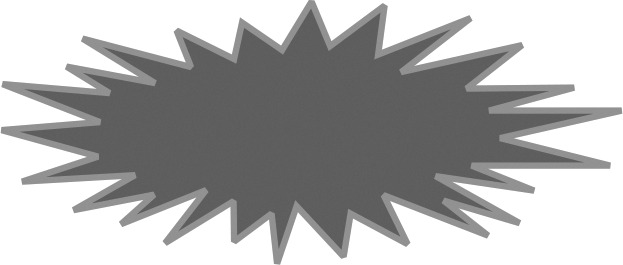
   = Kaizen Burst, referred to as a change indicator, signifies improvements planned for specific work processes, particularly in steps 5 and 7.


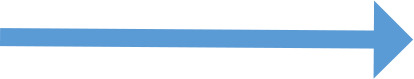
     = Manual Information is represented by an arrow indicating the flow of information, with personnel responsible for recording and transmitting data.


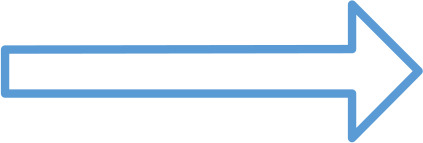
     = A Shipment Arrow. An arrow signifies drug transportation.


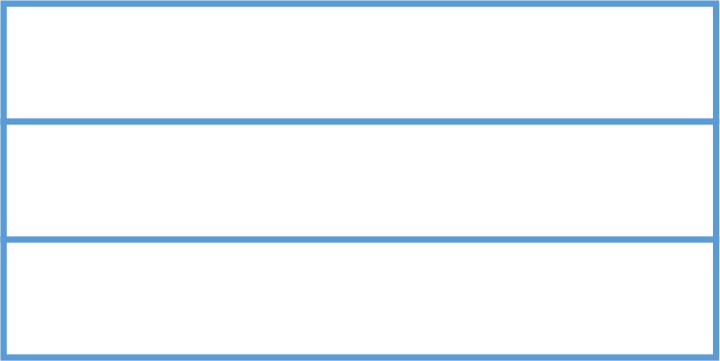
 = A Data Table is a display table that presents only Lead Time (LT) and Process Time (PT) data.


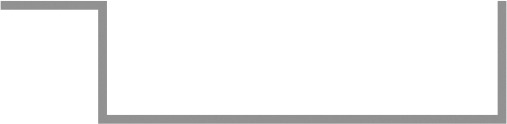
    = A Timeline Segment represents a segment of time. It shows the time at each step of the drug disbursement process using the lines. The line at the bottom represents Process Time, whereas the line at the top represents the Waiting Time.

Process Time (PT) refers to the operating time of a step per unit volume of work. In this study, one unit of work corresponds to withdrawing two medicine requisition slips once a week.

Waiting Time refers to the time between processes in the drug disbursement process.

Lead Time (LT) refers to the total time, which is the sum of the Process Time and Waiting Time.

### Value Stream Mapping

Value Stream Mapping comprises three main components. Here are the details:

Component 1: Work Flow

The current status of value stream mapping illustrates the flow of work through three departments: the drug warehouse (comparable to the supplier) and two drug warehouse sub-stocks (comparable to customers). The existing steps or activities are represented by Process Box symbols, each detailing the activities, personnel involved, and their roles. There are seven process boxes or steps, with steps 1-5 occurring in the drug warehouse and steps 6-7 in the two sub-stocks of the drug warehouse. The details of each step are as follows.

Step 1) A pharmacist receives a medicine requisition from the outpatient and inpatient medicine rooms at both locations. They reviewed the items and approved the quantity of each drug to be withdrawn in the subsequent step.
Step 2) A drug warehouse pharmacist delivers the approved drug requisition to a pharmaceutical assistant who deducts drug items from the system.
Step 3) Four drug warehouse employees arrange drugs according to the requirements of both warehouses.
Step 4) The pharmaceutical assistant checks the type and quantity of medicines prepared for withdrawal before delivery.
Step 5) Four drug warehouse employees transported the prepared drugs to both drug rooms and delivered them as withdrawn.
Step 6) A pharmaceutical assistant and six employees receive the drugs into the sub-stock warehouse, verify the quantity and list of medicines, and arrange them on the shelves once they are confirmed correct.
Step 7) Four pharmaceutical assistants in each medicine room inspect the medicines in the drug warehouse and withdraw drugs by completing a drug requisition slip, which is then delivered directly to the warehouse.

Component 2: Information Flow

The solid arrow symbolizes the flow of information and depict the transmission of data from one process to another. This includes information such as drug requisition forms and disbursement approval details. The flow of information is managed by staff who record and transmit data manually (Manual Information).

Component 3: Working period (Timeline)

A data table was employed when examining the process boxes in this study, This data table displays two key metrics. Process Time (PT) and Lead Time (LT), positioned at the bottom of the map. The timeline segment, located in the right corne is adjacent to the value stream. Below the diagram, the timeline comprises three components: the Total Lead Time, Total Process Time, and Activity Ratio. The Activity Ratio reflects the workflow efficiency. In an optimized value stream, this ratio should be higher, indicating that the time spent on productive processes outweighs system-wide time, thereby minimizing wasted time due to waiting or non-essential procedures.

For the drug disbursement process at the hospital, the current system utilizes timekeeping data gathered over one week of operation across all seven steps. The findings revealed a Total Process Time of 750 min, Total Lead Time of 1925 min, and Activity Ratio of 38.96 percent.

### Waste in the process

The workflow analysis results aim to identify activities that add value and contribute to waste within the system. Six types of waste were identified using the core concept of value analysis, as follows: Tabel 1.

**Table 1.** Analysis of Waste in the Current State of the Drug Disbursement Process within the Hospital.

Wastes	Details of the wastes in current state
1. Defects	- Writing medicine withdrawal requests without data on usage rates and approval without basic information, such as usage rates, results in dispensing medicines based solely on the amount allocated by the disbursement unit.
2. Transportation	- The drug transports from the drug warehouse to the drug warehouse sub-stock room occur multiple times due to the large quantity of drugs withdrawn in each round.
3. Excess Processing	-
4. Inventory	- There is a substantial quantity of medicine in both sub-stock drug warehouses.
5. Overproduction	- Medication withdrawals exceeding demand: Lack of information on actual usage rates prevents verification of the amount used.
6. Waiting	- Wait for disbursement once per week. - Arranged and checked medicines must wait for delivery the following day.
7. Motion	-
8. Non-Utilized Talent	- In each drug room, two pharmaceutical assistants are responsible for documenting drug withdrawals, categorized by the dosage form of the drug.

working period

From the analysis of activities divided according to their value, three types of work were identified:

Value Added (VA) activities involve process changes and add value.

Non-value-added (NVA) activities are unnecessary and considered waste that should be eliminated.

Necessary but non-value-added (NNVA) activities are necessary but do not add direct value and are challenging to change.

Here is a summary of the time spent on valuable and wasted activities in the work Table 2.

**Table 2.** Summary of Value/Waste and Duration of Activities in the Current Drug Disbursement Process within the Hospital.

Step	Activity	Value type	Wastes	Average time (minutes) *
1	Receive medication requisition and consider approval	VA	-	65
2	Deduct drug items from the inventory system	VA	-	80
3	Prepare drugs	VA	-	250
4	Repeat drugs check	NVA	-	1050
5	Prepare and deliver drugs	NVA	- Waiting - Moving	240
6	Receive drugs into the sub-stock warehouse and check the quantity and the list of medicines	NNVA	- Inventory	60
7	Check the items to be withdrawn, write a drug requisition slip, and deliver the drug withdrawal slip directly to the drug warehouse	NVA	- deficiency - excessive production - Not using human resources to their full potential - Waiting	180
**Total time in the system: 1,925 minutes.** **Time spent on valuable activities: 395 minutes** **Percentage of time spent on valuable activities: 20.52 %**				

NNVA = necessary but non-value-added, NVA = non-value-added, VA = value-added.

* Lead Time for each activity (lead time) is the Process Time combined with the Waiting Time.

The waste analysis revealed that waiting was the most significant waste in the system. Reviewing the current status, waiting occurs due to delays in receiving medical requisitions from both the outside and inside medicine rooms and waiting for the delivery of prepared medicines. These delays are exacerbated by incomplete information on the bills of lading and large quantities of medicines that need to be moved to the outside and inside medicine rooms. This results in excessive inventory and production as the reserves exceed actual needs, leading to unnecessary stockpiling. Additionally, human resources are not used to their full potential, as pharmacy officers must inspect medicines before disbursing them, adding to their workload. To address these issues, future work must be planned to eliminate waste and improve efficiency.

### Future work system

To review the current status, identify waste, and plan for its reduction while adding value to the drug supply inventory management system, we employed four key methods: 1) eliminating unnecessary parts, 2) combining several steps to save time and effort, 3) simplifying the process to avoid complexity and redundancy, and 4) using information management to replace manual tasks. The detailed actions based on these methods are as follows:

1. Eliminating Unnecessary Parts: To address unnecessary waiting periods, we identified that medication requirements from outpatient and inpatient medicine rooms should no longer be processed weekly. Instead, the drugs should be disbursed daily. This change aims to reduce waiting times and minimize the need for transportation from the warehouse to medicine rooms.
2. Combine Multiple Steps to Save Time: The current system requires drug warehouse employees to arrange medicines and then wait for officials to re-inspect them before the pharmaceutical assistant can dispense them. By switching to daily disbursements, we anticipate a reduction in the number of drug items and quantities. Consequently, the steps of organizing and checking can be combined. Drug warehouse employees and pharmaceutical assistants can complete the medication arrangement and inspection in the same step, thus eliminating the need for post-arrangement checks and reducing overall waiting time.
3. Simplifying the Process: To avoid complexity and redundancy in sending disbursement information to outpatient and inpatient medicine rooms, we will utilize electronic information transmission. By applying the concepts of the Kanban and vendor-managed inventory (VMI) systems, the approval process for drug disbursement will become more efficient and streamlined. This approach will ensure timely information delivery, making the disbursement and approval processes easier and reducing deficiencies in withdrawal information.
  The Kanban system is a lean concept tool used to control product inventory by maintaining low stock levels. This process is driven by customer demand, reducing costs and preventing overproduction to keep up with fluctuating needs ( Womack, J. P., et al., 1990). Electronic Kanban (e-Kanban) enhances this system by keeping it more up-to-date and timely, and managing just-in-time inventory based on actual demand ( Donnelly, G. T., et al., 2016).
  The VMI system is used to help reduce inventory and manage products to keep them moving according to the demand. Deliver raw materials that are made daily to order and can control delivery products that can withstand time can be combined ( Waller, M., et al., 1999).
4. Information management is essential for planning the implementation of the e-Kanban and VMI systems to manage pharmaceutical stockpiles effectively. These systems aim to reduce inventory levels and excessive production, ensuring that human resources are utilized for their full potential. A team of IT staff has begun developing an inventory management system in which the hospital is preparing to install it in drug warehouses and outpatient and inpatient medicine rooms. This system integrates the use of sub-stock warehouses by leveraging IT to retrieve daily drug dispensing data from outpatient and inpatient medicine rooms. This ensures timely information delivery to stockpile medical supplies every business day. In addition, barcode scanners will be installed at service points to facilitate the use of this new system, which will adjust drug delivery once per week to daily drug delivery by following up with the drug supply warehouse on the use of drugs in the service unit by extracting information on the number of medicines used each day from the HosXP program of the hospital's outpatient and inpatient medicine rooms.
  - The outpatient and inpatient medicine rooms pick up drugs from specified Kanban boxes. When the drug in the first box is gone, the employee in each room scans the barcode in the system to notify the drug warehouse of the drugs that have reached the reorder point each day. While waiting for replenishment, they used the drugs reserved in the second box.
  - The pharmacist at the drug warehouse checked the information in the system every morning to track the amount of medicine used daily in each medicine room. To confirm this information, if the drug room does not send data when the number of medicines reaches the reorder point, the pharmacist at the drug supply warehouse will verify with the medicine room to ensure accuracy. This allows for timely refilling of medicines before they are returned to the service unit.
  - When the amount of medicine used reaches a specified amount in the Kanban box, the pharmacist at the drug warehouse sends the information to the pharmaceutical assistant at the drug supply warehouse to record the withdrawal and dispense the drugs from the system. A bill of lading was printed for approval, and the information was sent to the medical staff according to the list of medicines needed by the service unit. Medicines are delivered in the afternoon every day.

The duty officer at the drug warehouse delivers the drugs and requisitions to the service unit. Drug warehouse staff then inspect and receive the drugs, placing them into specified Kanban boxes and updating the system accordingly. The medical supply inventory management system is designed for convenience and efficiency, with ongoing development aimed at reducing inventory costs by minimizing the drug reserves in each room. This approach addresses the deficiencies caused by incomplete or inaccurate information and reduces transportation costs by eliminating the need for multiple deliveries. It also reduces overproduction and wastage due to expired medicines, optimizing human resources in the service area. Weekly medication checks and withdrawals are streamlined and facilitated by the information system, which enhances speed and reduces the workload on the medicine rooms.

**Figure 2.**
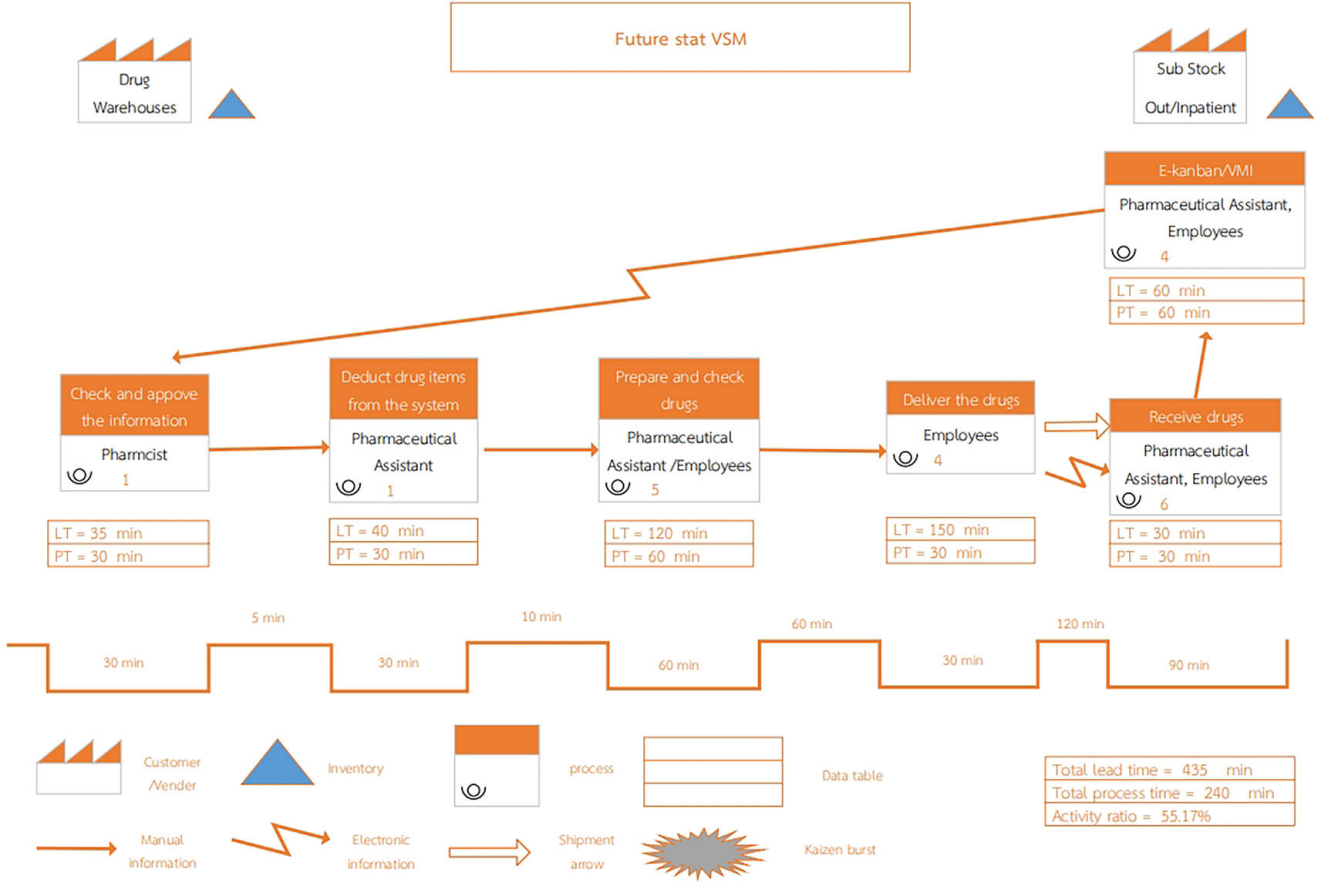
Future State Value Stream Mapping of inventory management system at Phukhieo Chaleomphrakeit Hospital.

Upon reviewing the current process, the future state value stream map has been streamlined from seven to six steps, as depicted in Figure 2 of the design. The future state outlines the following steps.

Step 1: The pharmacist in the drug warehouse processes and approves the daily drug withdrawal list each morning. Utilizing information from the e-Kanban and VMI systems, data are transmitted from the medicine rooms and the HosXp program without waiting for individual drug requisitions. This daily processing replaces the previous weekly schedul managed by a pharmacist in the warehouse.

Step 2: A pharmaceutical assistant updates the inventory control system to ensure accurate and complete medication withdrawal.

Step 3: Drug warehouse employees and pharmaceutical assistants arrange medicines according to the list and conduct inspections in a single step. This reduces the waiting times for reinspections and is managed by the entire warehouse team of five.

Step 4: Drug warehouse staff prepare and deliver medicines to both outside and inside medicine rooms daily, ensuring timely delivery and preventing excessive stockpiling. This process involves four staff members.

Step 5: Employees in each medicine room and pharmaceutical assistants inspect and receive medicines, ensuring appropriate inventory levels in both substock rooms. Six personnel were involved in this step.

Step 6: As medicines are picked up and used from the reserved stock in each medicine room, staff in both rooms retrieve medicines from specified Kanban boxes. Once the first box is empty, they scan barcode items in the system to notify the drug supply warehouse daily about medicines needing replenishment. They utilized the reserve in the second box while waiting for restocking. The pharmacist at the drug warehouse verifies daily medication usage and inventory levels, optimizes resource utilization and minimizes waiting times.

When adjusting the medical supply inventory management system, improvements were made to enhance efficiency, resulting in a reduced total lead time and total process time, as well as an increased activity ratio (activity ratio), as shown in Table 3.

**Table 3.** compares the results of drug inventory management before and after system development.

Performance	Current State	Future State	%Improvement
Total lead time (min)	1925	435	77.40%
Total process time (min)	750	240	68%
Activity ratio (%)	38.96	55.17	41.61%

- Total lead time: The sum of lead times across each step of the value chain process, measured in minutes.
  Current State: 65 + 80 + 250 + 1050 + 240 + 60 + 180 = 1925 minutes
  Future State: 35 + 40 + 120 + 150 + 30 + 60 = 435 minutes
- Total process time: The sum of process times across each step of the value chain process, measured in minutes.
  Current State: 60 + 60 + 240 + 90 + 60 + 60 + 180 = 750 minutes
  Future State: 30 + 30 + 60 + 30 + 30 + 60 = 240 minutes
- Activity ratio: Calculated using the formula:
  Activity ratio = (Total process time/Total lead time) x 100
  Current State: (750/1925) x 100 = 38.96%
  Future State: (240/435) x 100 = 55.17%

## Discussion

Action research was conducted following the concepts of Kemmis and McTaggart (1988), emphasizing public participation. The action research spiral involves four iterative steps: Planning, Action, Observation, and Reflection, with each cycle aiming to refine and enhance implementation plans through replanning.

Value Stream Mapping (VSM) serves as a pivotal tool for analyzing the drug disbursement process within the drug supply inventory management system. The initial phase involved planning in action research, where VSM was applied through Steps 1 to 3 ( Martin, K., & Osterling, M., 2014), focusing on designing future work systems to improve system efficiency.

Six types of waste were identified: defects, transportation issues, excess inventory, overproduction, waiting times, and the inefficient use of human resources. Proactive measures have been proposed to address these challenges.

1. Eliminating unnecessary steps by transitioning from paper-based to electronic medicine withdrawals to ensure accurate drug requisition and reduce deficiencies.
2. Streamlining operations by integrating organizational and inspection steps simplifies processes to eliminate redundancy and reduce waiting times.
3. Leveraging IT solutions to manage information efficiently, implementing programs based on e-Kanban and vendor-managed inventory (VMI) concepts to facilitate daily medicine disbursement. This adjustment aims to minimize waiting times and the need for multiple transportation trips, maintain appropriate inventory levels and optimize staff productivity.

By studying the current status of the drug inventory management system, Phukhieo Chaleomphrakeit Hospital was able to draw a map of the value stream in the future state, adjusting from seven to six steps. The total time spent in the current system was 1,925 min. However, when designing and planning the future state of the pharmaceutical inventory management system, it is expected that the total time spent in the system will be reduced to only 435 min, with the time spent on valuable activities reduced to 395 min. The percentage of time spent on valuable activities was 20.52%, which reduced waste while waiting. When adjusting the drug inventory management system, it was found that the efficiency of the system increased by reducing the total lead time by 77.40%, the total process time by 68%, and by increasing the activity ratio by 41.61%, which is consistent with previous studies.

In India, developing a supply chain value stream map (SCVSM) for government-sponsored drugs in the drug distribution system (DDS), according to VSM tool guidelines, reduced the total number of activities from 27 to 16 after implementing Kaizen in pharmaceutical distribution. The total product lead time decreased by 7.14%. Activities that did not add value included waiting-period transportation, improper processes, unnecessary inventory, and unnecessary movements. The study also proposed a future map free of these non-value-added steps, reducing the time it takes to deliver drugs from suppliers to patients, avoiding delays in the distribution of medicines, and reducing operating costs ( Dixit, A., et al., 2021).

A study conducted in Malaysia at the state-of-the-art Malaysian Government Pharmaceutical Organization found that the total processing time was reduced from 45,420 min to 11,940 min. This pharmaceutical case study demonstrates how VSM can identify key functions and eliminate unnecessary steps, highlighting the opportunities for ongoing enhancement in warehousing activities and the removal of process waste, including unnecessary inventory, equipment, labor, and storage space. Efforts have been focused on reducing excessive lead times for processes and products to enhance warehouse operations within the supply chain context. ( Abideen, A. Z., & Mohamad, F. B., 2019).

Examples of studies from various interventions with medication synchronization show that two steps in the process can be eliminated, resulting in a 69.4% reduction in packaging time in the workflow ( Renfro, C.P., et al., 2022). This case study, conducted in an emergency room, showed how VSM could identify bottlenecks and pain points in the patient care process, drawing a future diagram expected to reduce the total waiting time of the process and improve patient care by 3.7% from the beginning to the end of the process ( Cardoso, W., 2020). In addition, data were collected from healthcare systems in various countries with a review of relevant studies. The use of Value Stream Mapping (VSM) techniques to improve systems has shown positive effects on the time-related dimensions of process quality and outcomes. Specifically, VSM has been effective in reducing non-value-added time, such as waiting time, and various types of waste. ( Nowak, M. et al., 2017) ( Marin-Garcia, J.A., et al., 2021), ( Zdęba-Mozoła, A., et al., 2022).

In Thailand, a study on the use of VSM in health service processes, such as developing the drug disbursement process within Borabue Hospital, Maha Sarakham Province, aimed to find and reduce waste in the disbursement process. An analysis of all 10 work steps found only three valuable activities, with four activities that did not add value and three that did not add value but were necessary in the process. The trend in this study indicates a consistent direction. From this study, it is necessary to assess the current status to plan future work in managing the medical supply warehouse, designing work systems that reduce waste and adding value to make work more efficient.

In addition, the study was limited in terms of time, as only steps 1 to 3 of the VSM tool were performed out of the five steps. There should be continuous work in Step 4, which involves developing the transformation plan, creating a monitoring plan, and reporting the plan regularly to ensure continuous operations and improvement. Step 5, which focuses on achieving and sustaining change, must also be implemented. This includes regularly and continuously applying the PDSA (Plan Do Study Adjust) cycle. Real data should be collected according to the new method to observe the actual results and improve the new process to be suitable for the agency in the future.

Regarding potential improvements, it is expected that enhancing the satisfaction period concerning waiting time for those involved will likely increase sufficiency and availability, making work more convenient. The highlight of this study is the design of the work systems by those involved. This tool helps reduce waste and has been developed collaboratively to make operations run more smoothly.

## Conclusion

The study analyzed the process diagram of the drug inventory management system in all its steps using Value Stream Mapping (VSM), a key tool in Lean Healthcare principles. The VSM provides visibility to current workflows, facilitating the design of more efficient future systems. This study made significant improvements by identifying problems in the medical supply inventory and incorporating them into the design of the value stream map for a newly developed drug inventory management system. The cycle was adjusted from once a week to sending medicine to outpatient and inpatient medicine rooms on regular business days. This adjustment was expected to decrease the amount of medicine in the dispensary, with the treasury reserve rate now decreasing and trackable. Additionally, the pharmaceutical officer in the drug room no longer needs to survey the drugs and write requisitions according to the form submitted to the drug supply warehouse, thereby reducing storage space and minimizing waiting times. VSM, acts as a visualization tool for the supply chain and value stream, grounded in the Toyota production system, and significantly supports the effective implementation of lean methodologies. ( Teichgräber, U. K., & de Bucourt, M., 2012). This allows the team to see the flow of the work system, search for waste, and design future work systems that are more responsive and efficient.

### Limitations

Important principles of work system development using VSM include delivering valuable work to service recipients, primarily from the service recipient’s perspective. VSM is a simple and effective tool; however, this study had several limitations. First, the service recipients in this study were outpatients and inpatients in medicine and drug supply warehouses. These stakeholders may not have fully responded to all suggestions, such as having drug warehouse employees deliver and store medications in each room to reduce the workload for outpatient and inpatient medicine room employees. Additionally, the inspection process should involve staff from each room checking and receiving the delivered medicines from the medicine room for double checking. This can increase the workload in the medicine room, which requires daily storage. The study period was limited to developing and implementing a Future State. There has been no follow-up or regular improvement in work. Future studies should include additional planning to follow up on the work system development plan and continuously develop a plan for future state assessments. This clarifies whether valuable work can be delivered to service recipients. Indicators that clearly demonstrate the value of work are required to reduce waiting times and improve efficiency. The study's results are inconclusive but indicate the direction for application to increase efficiency. This study involved only a small group, reflecting limited perspectives, and the duration of the study was short. If a larger number of people were involved and the observation and discussion periods were extended, a wider variety of perspectives could be revealed, providing a broader view of the system.

### Suggestions

The design of the future state should be presented to the pharmaceutical and therapeutic committee to ensure consistency with the hospital's policy. This includes support for transportation, information, personnel, and other resources that are necessary to create an efficient new work system. The system should be capable of managing and monitoring the work for continuous development. There should be a structured approach to follow-up and report work results to the hospital's Pharmaceutical and Therapeutics Committee. Additionally, the PDSA (Plan Do Study Adjust) cycle should be implemented regularly and continuously. Improvements should be reported every six months to one year to ensure ongoing progress and adaptation.

## Ethics and consent

This research was approved by Center for Ethics the Human Research of Khon Kaen University on January 27, 2023, referene no. is HE652237. The researchers provided an explanation of the study and obtained informed consent from all participants involved in the discussion before conducting the study, using a written consent form. All participants were required to sign the consent form, which was approved by the ethics committee.

## Data Availability

Figshare: Application of Value Stream Mapping to Design and Develop an Inventory Management System in a Hospital. Doi: https://doi.org/10.6084/m9.figshare.28366301 ( Praewchit, R., 2025). This project contains the following underlying data: Requests for English excerpts are welcome for data in non-English languages.
• Focus Group Discussion Data Collection Form.docx • Calculation.docx • VSMcurrentandfuturestate.vsdx Data are available under the terms of the Creative Commons Attribution 4.0 International license (CC-BY 4.0).
