# Application of value stream mapping to design and develop an inventory management system in a hospital

**DOI:** 10.12688/f1000research.159736.2

**Published:** 2025-10-09

**Authors:** Praewchit Ritthaisong, Nusaraporn Kessomboon

**Affiliations:** 1 Khon Kaen University, Nai Mueang, Khon Kaen, Thailand

**Keywords:** Value Stream Mapping (VSM), Lean Management, Inventory Management System, Medical supply warehouse.

## Abstract

**Background:**

Insufficient drug reserves in hospitals pose significant challenges in management, procurement, and distribution. Rising demand often leads to shortages, while overstocking causes waste and expiration. These issues reflect inefficiencies in medical supply warehouse management and highlight the need for improvement. Therefore, enhanced resource management processes are essential to ensure a balanced and efficient medication reserve system. Lean Management is a systematic approach to eliminate waste, reduce costs, and improve efficiency. Value Stream Mapping (VSM), a core Lean tool, supports process redesign by categorizing activities as value-added (VA), necessary but non-value-added (NNVA), or non-value-added (NVA). In this study, VSM was employed to comprehensively analyze the drug disbursement process within the medical supply warehouse management system and to design and develop an enhanced drug inventory management system to improve efficiency and effectiveness.

**Method:**

This research employed action research methodology with 22 participants from the drug warehouse, outpatient, and inpatient medicine rooms. A user-centered system was planned through focus group discussions. This phase emphasized conceptual framework development and planning. Potential improvements were expected to enhance satisfaction, reduce waiting time, and increase convenience, sufficiency, and availability.

**Result:**

Phukhieo Chaleomphrakeit Hospital created a future state value stream map, reducing process steps from seven to six. The current system required 1,925 minutes. The redesigned system is expected to take 435 minutes, including 395 minutes for valuable activities. The proportion of value-added time increased to 20.52%, significantly reducing waste from waiting.

**Conclusion:**

The study demonstrates the effectiveness of VSM in identifying inefficiencies and redesigning processes. The collaboratively developed system eliminated unnecessary steps, reduced waiting times, and enhanced operational efficiency. This provides a practical model for improving pharmaceutical supply chain management in similar hospital contexts.

## Introduction

In hospital management, procurement, and distribution of medicines, several problems may arise, such as insufficient drug reserves ready for use, particularly in chronic disease medications or items that patients require continuously. For certain drugs with rapidly increasing demand, the reserve rate may be inadequate, leading to potential shortages. Consequently, hospitals often increase their stockpiles beyond normal levels, resulting in excessive inventory and a heightened risk of drug expiration. These issues reflect inefficiencies in medical supply warehouse management, highlighting the need for improvement to achieve greater effectiveness.

Lean Management is a systematic approach designed to eliminate waste, thereby reducing costs and enhancing efficiency, making it more effective than other management systems ( Lanza-León, P., et al., 2021) delineate this system based on five core principles.

• **Specify Value** – defining value from the service recipient’s perspective.
• **Identify the Value Stream** – analyzing value streams to detect and eliminate waste.
• **Flow** – ensuring processes are continuous and seamless.
• **Pull** – initiating processes based on demand or readiness of the next step.
• **Pursue Perfection** – striving for continuous improvement ( Womack & Jones, 1996, 2003; Lanza-León et al., 2021).

Since its application in the healthcare sector in 2009, Lean Management has demonstrated notable benefits, including reduced waiting times, decreased unnecessary hospital admissions, lower costs, and fewer errors. Moreover, thier adoption has bolstered the quality, safety, and efficiency of clinical processes ( Lanza-León, P., et al., 2021).

**Value Stream Mapping (VSM)** is a key Lean tool that creates visual maps of resource and information flows throughout the supply chain. It categorizes activities into three types: Value-Added (VA), Necessary but Non-Value-Added (NNVA), and Non-Value-Added (NVA) ( Martin & Osterling, 2014; Shararah, 2013). This classification enables the identification of waste points and redesign of processes to reduce time and increase efficiency. VSM plays a crucial role in healthcare, such as reducing waiting times and eliminating unnecessary steps, thereby improving patient-centered services and satisfaction ( Nowak et al., 2017). However, to ensure effectiveness, staff education and training are essential to reduce resistance and promote a culture of continuous improvement ( Gellad & Day, 2016). VSM has been applied in multiple industries including textiles, pharmaceuticals, food, telecommunications, and oil & gas to optimize indicators such as lead times, inventory levels, and overall efficiency ( Batwara et al., 2023; Seth & Gupta, 2005).

Studies have highlighted the use of VSM in various contexts:

• **Brazil**: VSM to analyze healthcare environments. The results of this study indicate that the proposed VSM model can identify bottlenecks in operations and various forms of waste, leading to insights into operational inefficiencies and bottlenecks ( Henrique et al., 2016).
• **
India**: A Supply Chain VSM (SCVSM) was developed for government sponsored drug distribution, reducing non-value-added activities. Following the VSM guidelines, future SCVSM activities were reduced from 27 to 16 after the Kaizen implementation, leading to a 7.14% reduction in the total product lead time ( Dixit et al., 2021).
• **Malaysia**: VSM in pharmaceutical warehousing reduced total processing time. The study revealed significant improvements: the total processing time decreased from 45,420 to 11,940 min, and the non-value-added time was reduced from 21,060 to 3,120 min. Workforce requirements also decreased from 51 to 31 (39.21%) ( Abideen & Mohamad, 2019).
• **Germany**: VSM to reduce waste in the procurement process of tube expander coils (endovascular stents). The current state analysis using the VSM identified 13 steps in the procurement process. Among these, only two steps were recognized as value-adding activities, where as five steps were identified as non-value-adding activities ( Teichgräber & de Bucourt, 2012).
• **United States**: VSM improved medication synchronization processes in pharmacies. As a result of these interventions, the two process steps were eliminated, leading to reduced packaging times. This demonstrates that Value Stream Mapping is a valuable tool for identifying and eliminating non-value-adding activities, thereby improving operational efficiency and standardization ( Renfro et al., 2022).
• Literature reviews indicate that most VSM applications occur in tertiary hospitals, with positive outcomes but lacking standardization and environmental sustainability indicators ( Batwara et al., 2023).

In Thailand, VSM has been applied in several hospitals. At **Nopparat Rajathanee Hospital**, VSM analysis of outpatient drug dispensing reduced average waiting times significantly. Before improvement, the average waiting time for receiving medicine was 54.01 ± 11.24 minutes. Following improvements facilitated by VSM, this waiting time decreased significantly to 46.11 ± 24.45 minutes ( Chanbancherd et al., 2012). Similarly, at Borabue Hospital in Mahasarakham, VSM was use to develop the drug disbursement process with the aim of identifying and reducing waste. Analysis of the ten work steps revealed that only three activities were value-adding, four activities were non-value-adding, and three were necessary but did not add value. Value-adding activities accounted for 30.60% of the total lead time, prompting the redesign of the work system to enhance efficiency ( Jitthananan, 2017; Jitthananan et al., 2017).

Phukhieo Chaleomphrakeit Hospital, located in Chaiyaphum Province, is a small general hospital (M1) with 300 beds that, serves the entire Phukhieo District. It also acts as a host hospital and network leader challenges include emergency shortages, expired drugs, and excessive reserves beyond optimal levels. As of October 2021, the hospital has managed a list of 588 items. There are two service units with sub-stock: outpatient and inpatient medicine rooms. Consequently, there have been instances of drug shortages despite the availability of drugs in warehouses, contributing to an increasing number of expired medicines. The medical disbursement process within the hospital has been identified as lengthy and complex, contributing to insufficient readiness for use and placing a disproportionate workload on available personnel. Analysis of current workflows has identified inefficiencies, prompting the development of a plan to implement new systems aimed at reducing waste and enhancing the efficiency of drug distribution.

VSM is thus recognized as an essential Lean Management tool for identifying and reducing waste, improving processes, and enhancing both patient and organizational outcomes. Its application requires multidisciplinary participation and fosters continuous improvement. Evidence from both domestic and international studies confirms that VSM can reduce waste, shorten waiting times, and improve drug management efficiency. Accordingly this study employed VSM to comprehensively analyze the drug disbursement process within the inventory management system, and identify existing waste and planning strategies for waste reduction. Designing a new system, such as the use of an e-Kanban system to enhance efficiency. The aim is to enhance the efficiency of the medical supply inventory management system.

This study aims to design and develop a new medical supply inventory management system that is efficient and effective by analyzing current inventory management processes to identify inefficiencies, applying Value Stream Mapping (VSM) as a framework for designing the new system, and improving processes to reduce waste and enhance system efficiency.

## Method

This research was conducted using action research methodology based on the concepts proposed by Kemmis and McTaggart (1988) and Kemmis, S., et al. (2014). Action research is characterized by its participatory approach, which aims to enhance the efficiency and effectiveness of operational practices by analyzing current conditions and addressing existing challenges ( Chatakarn, V., 2015). It involves active engagement in problem-solving within real-world contexts, fostering continuous improvement in operational quality ( Johnson, AP, 2012). Action research typically involves four iterative steps: Planning, Action, Observation, and Reflection. These steps form a cyclical process of self-reflection and iterative improvement, in which each cycle informs subsequent actions and refinements ( Kemmis, S., & McTaggart, R., 1988). In this study, Value Stream Mapping (VSM) ( Martin, K., & Osterling, M., 2014) was employed to comprehensively analyze the drug disbursement process within the medical supply warehouse management system. This phase primarily focused on the initial planning step, outlining the conceptual framework of the research.

### Participants

Purposive sampling was employed to ensure inclusion of all units directly involved in the drug requisition and disbursement system, thereby guaranteeing that the data accurately represented the actual workflow. Engage in discussions and collaborative planning with pharmaceutical warehouse staff to understand their challenges. Consult with key personnel from the drug warehouse, outpatient medicine room, and inpatient medicine room, involving 22 individuals.

• consisting of 6 staff members at the drug warehouse (1 pharmacist, 2 pharmaceutical officers, and 3 drug store staff )
• consisting of 16 personnel responsible for drug warehouse sub-stocks in each service unit (6 pharmacists, 4 pharmaceutical officers, and 6 pharmacy staff ).

The researchers provided an explanation of the study and obtained informed consent from all participants involved in the discussion before conducting the study, using a written consent form. All participants were required to sign the consent form, which was approved by the ethics committee. Gather insights into their operational needs and areas requiring improvement in medical warehouse management.

### Procedures and data collection

Plan the development of a user-centric system through focus group discussions aimed at identifying and prioritizing improvement opportunities. Conduct joint cause analysis sessions to effectively address the identified issues in routine operations.

Developed the Current State VSM by:

• Direct observation of every step in the drug requisition–disbursement process.
• Time measurement (stopwatch timing) for each activity to obtain Process Time (PT); Lead Time (LT) was computed as PT + Waiting Time; Activity Ratio was calculated as the proportion of value-added time to total time.
• Extracting quantitative data from requisition forms and the medical supply disbursement system

Quantitative data were collected, such as the number of transportations and waiting times. Six categories of waste were identified, namely Defects, Transportation, Inventory, Overproduction, Waiting, and Non-utilized Talent.

The current inventory management process was studied and analyzed comprehensively using Value Stream Mapping (VSM). The existing workflow across all stages was mapped to pinpoint inefficiencies and areas of improvement in drug supply warehouse operations.

### Future state design

Integrate findings from the VSM analysis into the design of a future state value stream map for an enhanced inventory management system by focus group discussions. The future work plan was designed with development strategies proposed by the researchers, incorporating consultation and input from relevant stakeholders who participated in focus group discussions. Propose adjustments, such as transitioning from weekly to daily medication delivery to outpatient and inpatient medicine rooms. This change aims to minimize inventory levels in the dispensary and enhance the traceability of drug reserves. Streamline processes between the drug supply warehouse and pharmaceutical assistants by eliminating manual surveys and requisition writing.

### Outcomes and measurements

• **Waste (time-based):** defined as non–value-added time occurring within each process step.
• **Process Time (PT):** measured directly on site via stopwatch timing and cross-checked with data from the electronic disbursement system.
• **Lead Time (LT):** calculated as the sum of PT and Waiting Time for each activity.
• **Activity Ratio:** determined by dividing value-added time by the total process time.

In cases where activity times varied across items, an aggregation rule was applied. Specifically, averages were calculated based on a standard number of requisition forms to ensure comparability across processes.

• Open-ended questions were employed to capture perceptions and experiences of participants, such as:
*“Does the supply system facilitate timely, simple communication for drug requisition and replacement?”*
*“How satisfied are you with the warehouse management system?”*

Prior to implementation, all open-ended questions were reviewed for content validity by a panel of three subject matter experts.

### Data analysis

**Qualitative data** (focus groups): analyzed by content analysis with coding and thematic categorization of issues.

**Quantitative data** (timings, inventory metrics): analyzed using descriptive statistics (means, percentages) and Current and Future State comparisons.

### Application of VSM per Martin & Osterling (2014)

The VSM tool involves five steps,

Step 1: Setting the Stage and Enabling Success

This step was conducted over a period of 1 week

• A team comprising six drug warehouse employees and 22 participants was involved in setting goals and understanding the existing inventory management system.
• Officials from the medical supply warehouse section, pharmacists, pharmaceutical officers, and staff from the outpatient and inpatient medicine rooms were included.
• Analyze operational problems by explaining workflows in each responsible section and plan initial data collection through observations.

Step 2: Understanding the Current State

This step was conducted over a period of 1 month

• Engage all 22 officials in surveying and observing operations, with drug warehouse staff focusing on prescription receipt for medicine delivery to outpatient and inpatient rooms.
• Record the observed operations and document the flow of activities from the drug warehouse to the substock of the medicine rooms.
• Study and analyze the process diagram of the current inventory management system across all steps, identifying operational issues and gathering improvement needs.
• Conduct focus group discussions to jointly analyze causes and propose solutions for routine work issues.
• Create a value stream map using Visio 2013 to visualize the workflow from the medical supply warehouse to the two medicine room sub-stocks.
• Discuss and address questions regarding process issues, identify waste steps, and establish timelines for each activity.

Step 3: Designing the Future State

This step was conducted over a period of 1 week

• Develop an efficient work system by eliminating unnecessary steps, simplifying processes, and utilizing information management to reduce personnel involvement in certain steps.

Step 4: Developing the Transformation Plan

Step 5: Achieving and Sustaining Transformation

In this study, steps 1-3 were utilized according to Martin and Osterling (2014) to design a future work system that enhances efficiency.

## Results

**Figure 1.**
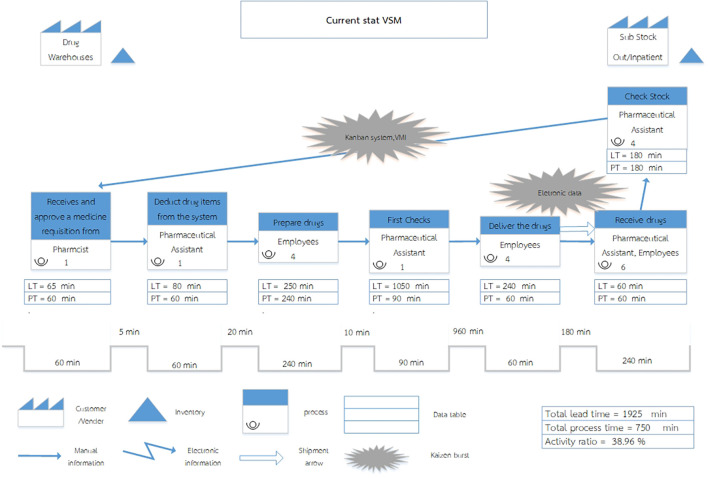
Current State Value Stream Mapping of the Inventory Management System at Phukhieo Chaleomphrakeit Hospital.


During the focus group discussions involving twenty-two staff members, several critical issues with the current medical supply inventory management system were identified. The primary concerns included the practice of stocking medicines weekly, which led to maintaining a high inventory value. This approach often results in shortages of essential medicines, necessitating emergency restocking between regular supply cycles. Furthermore, staffing shortages exacerbate the workload, particularly during peak times when large quantities of drugs are dispensed. The inefficiencies caused by these practices were evident in the significant amount of manpower and time required to store drugs in the warehouse. Moreover, the weekly disbursement schedule created spikes in workload, increasing the risk of medication errors and straining the available storage space. These findings underscore the need to redesign the inventory management system to effectively address these operational challenges ( Figure 1).

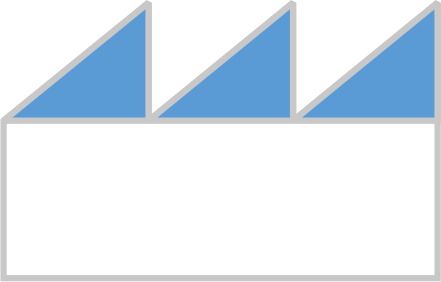
 = The Customer/Supplier relationship defines the drug warehouse as the supplier currently serving as the service provider for dispensing drugs to customers, namely the two medicine rooms.


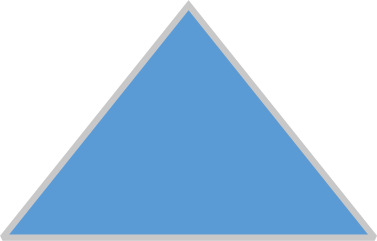
 = Inventory, refers to medicines held in reserve, encompassing drug warehouses, outpatient medicine rooms, and inpatient medicine rooms.


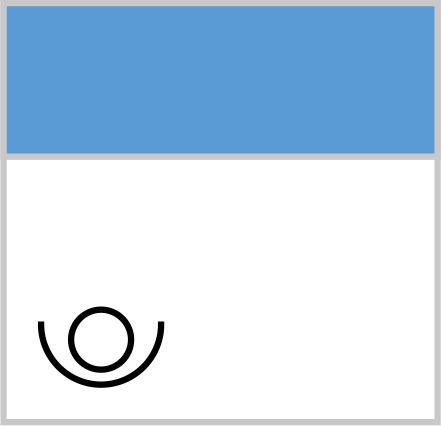
 = In the current state, the process depicts each step in the workflow of the system, which comprises seven steps.


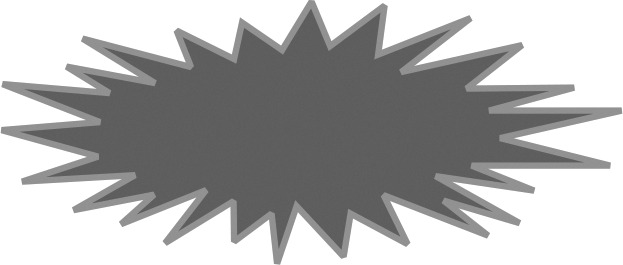
 = Kaizen Burst, referred to as a change indicator, signifies improvements planned for specific work processes, particularly in steps 5 and 7.


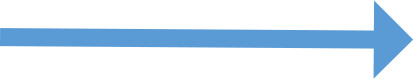
 = Manual Information is represented by an arrow indicating the flow of information, with personnel responsible for recording and transmitting data.


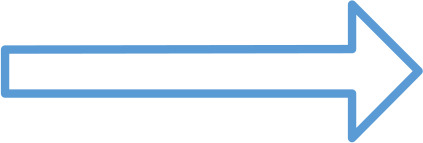
 = A Shipment Arrow. An arrow signifies drug transportation.


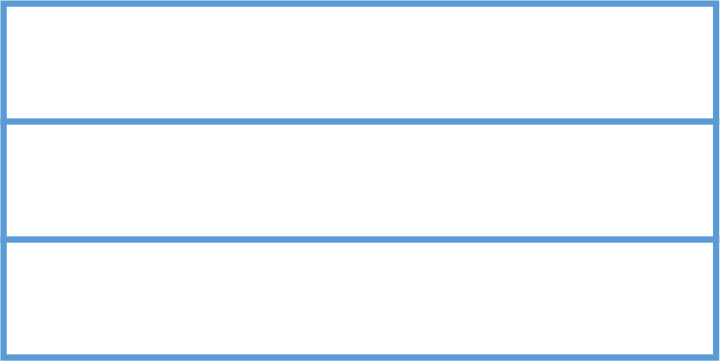
 = A Data Table is a display table that presents only Lead Time (LT) and Process Time (PT) data.


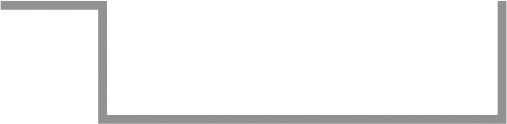
 = A Timeline Segment represents a segment of time. It shows the time at each step of the drug disbursement process using the lines. The line at the bottom represents Process Time, whereas the line at the top represents the Waiting Time.

Process Time (PT) refers to the operating time of a step per unit volume of work. In this study, one unit of work corresponds to withdrawing two medicine requisition slips once a week.

Waiting Time refers to the time between processes in the drug disbursement process.

Lead Time (LT) refers to the total time, which is the sum of the Process Time and Waiting Time.

The Current State Value Stream Map (VSM) encompasses three principal components: work flow, information flow, and time dimension. In its present configuration, the system consists of seven sequential processes, beginning with requisition receipt, approval, drug preparation, verification, and transportation, followed by drug receipt at the satellite pharmacies and the subsequent preparation of new requisition forms. The majority of these activities are undertaken within the supply warehouse, while certain processes are carried out at the sub-stcok in outpatient medicine rooms, and inpatient medicine rooms.

### Waste in the process

The workflow analysis results aim to identify activities that add value and contribute to waste within the system. Six types of waste were identified using the core concept of value analysis, as follows: Tabel 1.

**Table 1.** Analysis of Waste in the Current State of the Drug Disbursement Process within the Hospital.

Wastes	Details of the wastes in current state
1. Defects	- Writing medicine withdrawal requests without data on usage rates and approval without basic information, such as usage rates, results in dispensing medicines based solely on the amount allocated by the disbursement unit.
2. Transportation	- The drug transports from the drug warehouse to the drug warehouse sub-stock room occur multiple times due to the large quantity of drugs withdrawn in each round.
3. Excess Processing	-
4. Inventory	- There is a substantial quantity of medicine in both sub-stock drug warehouses.
5. Overproduction	- Medication withdrawals exceeding demand: Lack of information on actual usage rates prevents verification of the amount used.
6. Waiting	- Wait for disbursement once per week. - Arranged and checked medicines must wait for delivery the following day.
7. Motion	-
8. Non-Utilized Talent	- In each drug room, two pharmaceutical assistants are responsible for documenting drug withdrawals, categorized by the dosage form of the drug.

Here is a summary of the time spent on valuable and wasted activities in the work Table 2.

**Table 2.** Summary of Value/Waste and Duration of Activities in the Current Drug Disbursement Process within the Hospital.

Step	Activity	Value type	Wastes	Process time *	Waiting time *
1	Receive medication requisition and consider approval	VA	-	60	5
2	Deduct drug items from the inventory system	VA	-	60	20
3	Prepare drugs	VA	-	240	10
4	Repeat drugs check	NVA	-	90	960
5	Prepare and deliver drugs	NVA	- Waiting - Moving	60	180
6	Receive drugs into the sub-stock warehouse and check the quantity and the list of medicines	NNVA	- Inventory	60	-
7	Check the items to be withdrawn, write a drug requisition slip, and deliver the drug withdrawal slip directly to the drug warehouse	NVA	- deficiency - excessive production - Not using human resources to their full potential - Waiting	180	-
**Total time in the system: 1,925 minutes.** **Time spent on valuable activities: 395 minutes.** **Percentage of time spent on valuable activities: 20.52%.**					

NNVA = necessary but non-value-added, NVA = non-value-added, VA = value-added.

* Lead Time for each activity (lead time) is the Process Time combined with the Waiting Time.

**Table 3.** Compares the results of drug inventory management before and after system development.

Performance	Before	After	%Improvement
Total lead time (min)	1925	435	77.40%
Total process time (min)	750	240	68%
Activity ratio (%)	38.96	55.17	41.61%

The waste analysis revealed that waiting was the most significant waste in the system. Reviewing the current status, waiting occurs due to delays in receiving medical requisitions from both the outside and inside medicine rooms and waiting for the delivery of prepared medicines. These delays are exacerbated by incomplete information on the bills of lading and large quantities of medicines that need to be moved to the outside and inside medicine rooms. This results in excessive inventory and production as the reserves exceed actual needs, leading to unnecessary stockpiling. Additionally, human resources are not used to their full potential, as pharmacy officers must inspect medicines before disbursing them, adding to their workload. To address these issues, future work must be planned to eliminate waste and improve efficiency.

### Proposed work system

To review the current status, identify waste, and plan for its reduction while adding value to the drug supply inventory management system. The future work plan was designed with development strategies proposed by the researchers, incorporating consultation and input from relevant stakeholders who participated in focus group discussions. We employed four key methods: 1) eliminating unnecessary parts, 2) combining several steps to save time and effort, 3) simplifying the process to avoid complexity and redundancy, and 4) using information management to replace manual tasks. The detailed actions based on these methods are as follows:

1. Eliminating Unnecessary Parts: To address unnecessary waiting periods, we identified that medication requirements from outpatient and inpatient medicine rooms should no longer be processed weekly. Instead, the drugs should be disbursed daily. This change aims to reduce waiting times and minimize the need for transportation from the warehouse to medicine rooms.
2. Combine Multiple Steps to Save Time: The current system requires drug warehouse employees to arrange medicines and then wait for officials to re-inspect them before the pharmaceutical assistant can dispense them. By switching to daily disbursements, we anticipate a reduction in the number of drug items and quantities. Consequently, the steps of organizing and checking can be combined. Drug warehouse employees and pharmaceutical assistants can complete the medication arrangement and inspection in the same step, thus eliminating the need for post-arrangement checks and reducing overall waiting time.
3. Simplifying the Process: To avoid complexity and redundancy in sending disbursement information to outpatient and inpatient medicine rooms, we will utilize electronic information transmission. By applying the concepts of the Kanban and vendor-managed inventory (VMI) systems, the approval process for drug disbursement will become more efficient and streamlined. This approach will ensure timely information delivery, making the disbursement and approval processes easier and reducing deficiencies in withdrawal information.
The Kanban system is a lean concept tool used to control product inventory by maintaining low stock levels. This process is driven by customer demand, reducing costs and preventing overproduction to keep up with fluctuating needs ( Womack, J. P., et al., 1990). Electronic Kanban (e-Kanban) enhances this system by keeping it more up-to-date and timely, and managing just-in-time inventory based on actual demand ( Donnelly, G. T., et al., 2016).
The VMI system is used to help reduce inventory and manage products to keep them moving according to the demand. Deliver raw materials that are made daily to order and can control delivery products that can withstand time can be combined ( Waller, M., et al., 1999).
4. Information management is essential for planning the implementation of the e-Kanban and VMI systems to manage pharmaceutical stockpiles effectively. These systems aim to reduce inventory levels and excessive production, ensuring that human resources are utilized for their full potential. A team of IT staff has begun developing an inventory management system in which the hospital is preparing to install it in drug warehouses and outpatient and inpatient medicine rooms.

**Figure 2.**
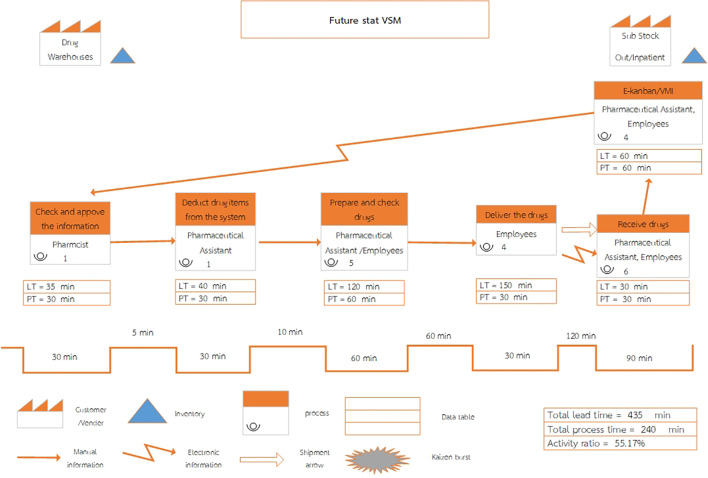
Future State Value Stream Mapping of inventory management system at Phukhieo Chaleomphrakeit Hospital.

Upon reviewing the current process, the future state value stream map has been streamlined from seven to six steps, as shown in Figure 2.

When adjusting the medical supply inventory management system, improvements were made to enhance efficiency, resulting in a reduced total lead time and total process time, as well as an increased activity ratio (activity ratio), as shown in Table 3.

• Total lead time: The sum of lead times across each step of the value chain process, measured in minutes.
• Total process time: The sum of process times across each step of the value chain process, measured in minutes.
• Activity ratio: Calculated using the formula:
• Activity ratio = (Total process time/Total lead time) × 100

## Discussion

By studying the current status of the drug inventory management system, Phukhieo Chaleomphrakeit Hospital was able to draw a map of the value stream in the future state, adjusting from seven to six steps. The total time spent in the current system was 1,925 min. However, when designing and planning the future state of the pharmaceutical inventory management system, it is expected that the total time spent in the system will be reduced to only 435 min, with the time spent on valuable activities reduced to 395 min. The percentage of time spent on valuable activities was 20.52%, which reduced waste while waiting. When adjusting the drug inventory management system, it was found that the efficiency of the system increased by reducing the total lead time by 77.40%, the total process time by 68%, and by increasing the activity ratio by 41.61%, which is consistent with previous studies. Six types of waste were identified: defects, transportation issues, excess inventory, overproduction, waiting times, and the inefficient use of human resources.

These findings are consistent with international studies. In India, the application of Supply Chain Value Stream Mapping (SCVSM) reduced the number of activities in the drug distribution system from 27 to 16, with total waiting time decreasing by 7.14% ( Dixit et al., 2021). In Malaysia, the total process duration was reduced from 45,420 minutes to 11,940 minutes, confirming the effectiveness of VSM at the system level ( Abideen & Mohamad, 2019). Similarly, eliminating two steps in the process can be eliminated, resulting in a 69.4% reduction in packaging time in the workflow ( Renfro et al., 2022), total waiting time of the process and improve patient care by 3.7% from the beginning to the end of the process in emergency departments ( Cardoso, W., 2020).

In addition, data were collected from healthcare systems in various countries with a review of relevant studies. The use of Value Stream Mapping (VSM) techniques to improve systems has shown positive effects on the time-related dimensions of process quality and outcomes. Specifically, VSM has been effective in reducing non-value-added time, such as waiting time, and various types of waste ( Nowak, M., et al., 2017; Marin-Garcia, J.A., et al., 2021; Zdęba-Mozoła, A., et al., 2022).

In Thailand, limited studies exist, but an investigation at Borabu Hospital, Maha Sarakham Province, aimed to find and reduce waste in the disbursement process. An analysis of all 10 work steps found only three valuable activities, with four activities that did not add value and three that did not add value but were necessary in the process. The trend in this study indicates a consistent direction. From this study, it is necessary to assess the current status to plan future work in managing the medical supply warehouse, designing work systems that reduce waste and adding value to make work more efficient. This finding is consistent with previous studies and further reinforces the existing evidence. Given the limited number of studies conducted in Thailand, this research provides an additional contribution by highlighting the benefits of applying VSM. And this study has planned the use of an e-Kanban system for future system development, which represents an additional contribution beyond previous research.

In this study, VSM was applied as a tool to analyze the overall drug distribution process, corresponding to the planning stage of action research. The research covered steps one through three of the VSM methodology ( Martin & Osterling, 2014), focusing on the design of a future system to enhance efficiency.

There should be continuous work in Step 4, which involves developing the transformation plan, creating a monitoring plan, and reporting the plan regularly to ensure continuous operations and improvement. Step 5, which focuses on achieving and sustaining change, must also be implemented. This includes regularly and continuously applying the PDSA (Plan Do Study Adjust) cycle.

Regarding potential improvements, it is expected that enhancing the satisfaction period concerning waiting time for those involved will likely increase sufficiency and availability, making work more convenient. Through the application of the system, the highlight of this study is the design of the work systems by those involved. This tool helps reduce waste and has been developed collaboratively to make operations run more smoothly. For continuous development, once the system design and development plan has been established, it should be implemented in practice with designated responsibilities and clear communication to *all those involved.* The system should be designed to facilitate ease of use by integrating IT participation to simplify operations. Moreover, continuous monitoring and evaluation of outcomes are required, with the results systematically reflected back to involved at different levels to inform future planning, adjustments, and improvements.

### Limitations

Important principles of work system development using VSM include delivering valuable work to service recipients, primarily from the service recipient’s perspective. VSM is a simple and effective tool; however, this study had several limitations. First, the service recipients in this study were outpatients and inpatients in medicine and drug supply warehouses. These stakeholders may not have fully responded to all suggestions, such as having drug warehouse employees deliver and store medications in each room to reduce the workload for outpatient and inpatient medicine room employees. Additionally, the inspection process should involve staff from each room checking and receiving the delivered medicines from the medicine room for double checking. This can increase the workload in the medicine room, which requires daily storage. The study period was limited to developing and implementing a Future State. There has been no follow-up or regular improvement in work. Future studies should include additional planning to follow up on the work system development plan and continuously develop a plan for future state assessments. This clarifies whether valuable work can be delivered to service recipients. Indicators that clearly demonstrate the value of work are required to reduce waiting times and improve efficiency. The study's results are inconclusive but indicate the direction for application to increase efficiency. This study involved only a small group, reflecting limited perspectives, and the duration of the study was short. If a larger number of people were involved and the observation and discussion periods were extended, a wider variety of perspectives could be revealed, providing a broader view of the system. Clear and detailed communication with all those involved, is essential when implementing changes in the management system. Regular supervision, consultation, and observation are necessary to ensure correct practice and to address potential resistance or fear of new methods arising from limited knowledge or understanding.

### Suggestions

The design of the future state should be presented to the pharmaceutical and therapeutic committee to ensure consistency with the hospital's policy. This includes support for transportation, information, personnel, and other resources that are necessary to create an efficient new work system. The system should be capable of managing and monitoring the work for continuous development. There should be a structured approach to follow-up and report work results to the hospital's Pharmaceutical and Therapeutics Committee. Additionally, the PDSA (Plan Do Study Adjust) cycle should be implemented regularly and continuously. Improvements should be reported every six months to one year to ensure ongoing progress and adaptation.

The system should be applied to other supply warehouses, including those in same hospitals as well as sub-district health promotion hospitals within the network. As this study required only a modest budget, it may be particularly suitable for hospitals at the initial stage of system development. Identified issues were reflected back to relevant departments to plan for further solutions. Following implementation, development outcomes, along with challenges and obstacles, to ensure that the findings could guide improvements in the new system processes and serve as a foundation for further development. Moreover, future studies should also record additional contextual factors that may influence system effectiveness, such as changes in hospital drug policies or staff turnover and rotations of new physicians.

## Conclusion

The study analyzed the process diagram of the drug inventory management system in all its steps using Value Stream Mapping (VSM), a key tool in Lean Healthcare principles. The VSM provides visibility to current workflows, facilitating the design of more efficient future systems. This study made significant improvements by identifying problems in the medical supply inventory and incorporating them into the design of the value stream map for a newly developed drug inventory management system. The cycle was adjusted from once a week to sending medicine to outpatient and inpatient medicine rooms on regular business days. This adjustment was expected to decrease the amount of medicine in the dispensary, with the treasury reserve rate now decreasing and trackable. Additionally, the pharmaceutical officer in the drug room no longer needs to survey the drugs and write requisitions according to the form submitted to the drug supply warehouse, thereby reducing storage space and minimizing waiting times. VSM, acts as a visualization tool for the supply chain and value stream. This allows the team to see the flow of the work system, search for waste, and design future work systems that are more responsive and efficient.

### Ethics and consent

This research was approved by Center for Ethics the Human Research of Khon Kaen University on January 27, 2023, referene no. is HE652237. The researchers provided an explanation of the study and obtained informed consent from all participants involved in the discussion before conducting the study, using a written consent form. All participants were required to sign the consent form, which was approved by the ethics committee.

## Data availability

Figshare: Application of Value Stream Mapping to Design and Develop an Inventory Management System in a Hospital. Doi: https://doi.org/10.6084/m9.figshare.28366301 ( Praewchit, R., 2025).

This project contains the following underlying data:

Requests for English excerpts are welcome for data in non-English languages.

• Focus Group Discussion Data Collection Form.docx
• Calculation.docx
• VSMcurrentandfuturestate.vsdx

Data are available under the terms of the Creative Commons Attribution 4.0 International license (CC-BY 4.0).
