# Supplementary material for: Application of value stream mapping to design and develop an inventory management system in a hospital
Source: F1000Res. 2026 Apr 15;14:365. Originally published 2025 Mar 31. [Version 3] doi: 10.12688/f1000research.159736.3 (PMC13142668; doi:10.12688/f1000research.159736.3)
Supplement: Peer review history

# Reviewer report: Reviewer response for version 2

**DOI:** 10.5256/f1000research.189130.r447435

**Published:** 2026-02-04

**Authors:** Aamir Rashid

**Affiliations:** 1 City University of New York (CUNY), New York, New York, USA

Thank you for your research on this interesting topic. While the manuscript is thoughtfully developed, a thorough reading has revealed several concerns that warrant critical attention.

1. The manuscript relies on a single-site action research case without a clearly articulated theoretical contribution beyond well-established Lean and VSM principles. While the operational improvements are quantified, the study remains confirmatory, reiterating known benefits of VSM rather than extending theory in healthcare operations or supply chain management.
2. The research design lacks rigor in inference. Outcomes are framed as “expected” improvements based on future-state mapping rather than empirically validated post-implementation results, which weakens causal claims. The absence of longitudinal follow-up or comparative benchmarking limits internal validity and generalizability.
3. Conceptually, “waste” is narrowly operationalized as time-based inefficiency, without engagement with inventory cost, service-level risk, or patient-safety trade-offs. This constrains the managerial and policy relevance of the findings. Moreover, the linkage between identified problems (shortages and expiries) and measured outcomes is indirect and insufficiently theorized.
4. The manuscript’s scholarly positioning is modest. It draws heavily on applied healthcare and Lean literature but does not engage deeply with core supply chain, operations management, or inventory theory debates, limiting its contribution to a largely practice-oriented audience. The paper is suitable as a local applied case, but its contribution remains incremental rather than field-advancing.
5. Methodologically, the study confuses process redesign with performance validation. Most reported improvements are expected or projected based on future-state mapping rather than empirically observed post-implementation outcomes. This undermines causal inference and weakens claims of effectiveness. The absence of a before–and–after comparison using realized data, or any counterfactual logic, is a major limitation.
6. The action research design is under-specified. The roles of participants, decision authority, and potential bias introduced through participatory mapping are not critically discussed. The focus group process is described procedurally but not analytically, leaving uncertainty about how consensus was reached or how conflicting perspectives were handled.

Is the work clearly and accurately presented and does it cite the current literature?

Partly

If applicable, is the statistical analysis and its interpretation appropriate?

Not applicable

Are all the source data underlying the results available to ensure full reproducibility?

No

Is the study design appropriate and is the work technically sound?

Partly

Are the conclusions drawn adequately supported by the results?

Partly

Are sufficient details of methods and analysis provided to allow replication by others?

No

Reviewer Expertise:

Supply Chain Management

I confirm that I have read this submission and believe that I have an appropriate level of expertise to confirm that it is of an acceptable scientific standard, however I have significant reservations, as outlined above.

# Response

**Published:** 2026-03-25

**Authors:** Praewchit Ritthaisong

**Affiliations:** Khon Kaen University, Nai Mueang, Khon Kaen, Thailand

Dear Reviewer,

 We sincerely thank you for your careful and insightful review. Your comments have been extremely valuable in improving the scientific clarity, methodological transparency, and theoretical positioning of our manuscript. Below we address each concern in detail.

 Comment 1: Limited theoretical contribution beyond Lean/VSM principles

 Response:

 Thank you for this important comment. We clarified that the study aims to extend the application of VSM to hospital pharmaceutical inventory management within a resource-constrained healthcare context. Additional discussion has been incorporated to position the study within healthcare operations and implementation science, emphasizing contextual adaptation, multidisciplinary participation, and integration with medication safety and shortage prevention.

 Comment 2: Outcomes are projected rather than empirically validated

 Response:

 We agree that future-state mapping represents expected performance. The manuscript has been revised to clearly distinguish between “projected improvement” and “observed outcomes”. We removed causal wording and now describe the findings as process redesign potential. We also added a statement in the limitations that full effectiveness requires post-implementation monitoring and longitudinal evaluation.

 Comment 3: Narrow conceptualization of waste

 Response:

 Additional explanation has been inserted in the Discussion linking process delay to medication availability risk and service continuity in hospital pharmacy systems.

 Comment 4: Limited engagement with supply chain and inventory theory

 Response:

 Thank you for this insightful comment. We agree that the manuscript primarily presents an applied case study. To strengthen the scholarly positioning, we have revised the Discussion section to better connect the findings with inventory management and supply chain management concepts. Specifically, the study now highlights how VSM-based redesign supports inventory control through reduced replenishment intervals, improved inventory visibility, and sub-stock management. These elements relate to core inventory management principles

 Comment 5: Lack of before-after comparison and causal inference

 Response:

 Thank you for this important comment. We clarified that the study is a process design and improvement study rather than an effectiveness evaluation. Accordingly, claims of effectiveness have been moderated throughout the manuscript, and a limitation statement has been added to emphasize that causal impact requires evaluation after implementation using realized data.

 Comment 6: Action research roles and focus group analysis unclear

 Response:

 We added details describing participant roles, decision authority, and consensus process. We also described how disagreements were resolved using structured facilitation and iterative validation sessions to reduce bias.

 We believe these revisions significantly improve the rigor, transparency, and positioning of the manuscript. We are grateful for your constructive feedback which has strengthened the study.

 Kind regards,

 Praewchit Ritthaisong

# Reviewer report: Reviewer response for version 2

**DOI:** 10.5256/f1000research.189130.r422120

**Published:** 2026-02-03

**Authors:** Puree Anantachoti

**Affiliations:** 1 Chulalongkorn University, Bangkok, Bangkok, Thailand

I am satisfied with the revisions made to the manuscript. Given the improvements, I recommend that this manuscript be accepted for indexing.

Is the work clearly and accurately presented and does it cite the current literature?

Partly

If applicable, is the statistical analysis and its interpretation appropriate?

No

Are all the source data underlying the results available to ensure full reproducibility?

Partly

Is the study design appropriate and is the work technically sound?

Partly

Are the conclusions drawn adequately supported by the results?

Yes

Are sufficient details of methods and analysis provided to allow replication by others?

No

Reviewer Expertise:

Social pharmacy, Administrative pharmacy, Health and Pharmacy policy, Access to medicine, Regulatory Sciences

I confirm that I have read this submission and believe that I have an appropriate level of expertise to confirm that it is of an acceptable scientific standard.

# Response

**Published:** 2026-02-15

**Authors:** Praewchit Ritthaisong

**Affiliations:** Khon Kaen University, Nai Mueang, Khon Kaen, Thailand

Dear Reviewer,

 Thank you very much for your time and for carefully reviewing our manuscript.

 We sincerely appreciate your positive evaluation and your recommendation that the manuscript is suitable for indexing. Your feedback has been very valuable in improving the clarity and quality of our work.

 We are grateful for your expertise and thoughtful assessment, and we hope our study will contribute to the field of hospital pharmacy operations and inventory management.

 Kind regards,

 Praewchit Ritthaisong

# Reviewer report: Reviewer response for version 2

**DOI:** 10.5256/f1000research.189130.r422121

**Published:** 2026-01-02

**Authors:** Rungpetch Sakulbumrungsil

**Affiliations:** 1 College of Pharmacy Administration, Thailand, Thailand; 2 Chulalongkorn University, Pathumwan, Thailand

Thank you for the revised manuscript and the detailed responses to the previous review. The revisions have improved the clarity of the introduction and strengthened the overall narrative coherence. However, several issues remain that should be addressed before the manuscript can be considered for indexing.

 1. Clarification and Explicit Statement of Research Objectives

 The overarching aim of the study is to develop a new inventory management system. To ensure consistency across the manuscript, the research objectives should be stated explicitly and concisely. I recommend formulating them as follows:

 1.    To analyze the current inventory management workflow using Value Stream Mapping (VSM) in order to identify inefficiencies.

 2.    To design a future-state inventory management system based on the findings from the workflow analysis.

 Presenting the objectives clearly will improve alignment between the Introduction, Methods, and Conclusion sections.

 2. Strengthen the Conclusion Section

 The conclusion should more clearly highlight two essential points:

 •    The redesigned system is expected to improve efficiency based on the projected reductions in lead time and process time.

 •    The actual impact of the redesigned system will depend on full implementation and subsequent monitoring, including follow-up evaluation and iterative improvement cycles.

 Explicitly acknowledging these points will strengthen the study’s implications and appropriately frame the scope of the current findings.

 3. Inclusion of a Conceptual Workflow Figure

 To enhance reader understanding of the research process, please consider adding a conceptual figure summarizing the main steps of the study. A simple diagram illustrating the progression

 Current State → VSM Analysis → Redesign Process → Future State

 would provide a helpful visual overview of the methodology, particularly for readers who are less familiar with Lean or action research approaches.

 4. Review for Grammatical Completeness and Coherent Sentence Structure

 To ensure readability and academic quality, I recommend that the authors conduct a thorough, manuscript-wide revision focusing on:

 •    grammatical accuracy,

 •    sentence completeness,

 •    clarity and coherence of ideas, and

 •    consistency in terminology across sections.

 A full-language review will help improve the overall clarity and professionalism of the paper.

 Overall Recommendation: Revisions Required

 This manuscript addresses an important issue in hospital pharmacy operations and offers a practical application of VSM within a real-world healthcare setting. With the additional revisions noted above, the manuscript will be further strengthened and more suitable for indexing.

Is the work clearly and accurately presented and does it cite the current literature?

Yes

If applicable, is the statistical analysis and its interpretation appropriate?

Yes

Are all the source data underlying the results available to ensure full reproducibility?

No source data required

Is the study design appropriate and is the work technically sound?

Yes

Are the conclusions drawn adequately supported by the results?

Yes

Are sufficient details of methods and analysis provided to allow replication by others?

Yes

Reviewer Expertise:

inventory management

I confirm that I have read this submission and believe that I have an appropriate level of expertise to confirm that it is of an acceptable scientific standard, however I have significant reservations, as outlined above.

# Response

**Published:** 2026-03-25

**Authors:** Praewchit Ritthaisong

**Affiliations:** Khon Kaen University, Nai Mueang, Khon Kaen, Thailand

Dear Reviewer,

 We sincerely thank you for your constructive comments and for recognizing the improvements made in the revised manuscript. Your suggestions have been very helpful in further strengthening the clarity and consistency of our work. Below we address each point in detail.

 Comment 1: Clarification of research objectives

 Response:

 The research objectives have now been explicitly stated in the Introduction section and aligned across the manuscript. The objectives are now presented as:

 (1) To analyze the current inventory management workflow using Value Stream Mapping (VSM) to identify inefficiencies, and

 (2) To design a future-state inventory management system based on the workflow analysis findings.

 Comment 2: Strengthening the conclusion section

 Response:

 The Conclusion has been revised to clearly distinguish between projected efficiency improvement and actual impact after implementation. We now explicitly state that the redesigned system is expected to improve efficiency based on reductions in lead time and process time, while acknowledging that real-world effectiveness depends on full implementation and continuous monitoring and improvement cycles.

 Comment 3: Addition of conceptual workflow figure

 Response:

 A conceptual workflow figure illustrating the research process (Current State → VSM Analysis → Redesign Process → Future State) has been added to the introduction section to improve reader understanding of the study design.

 Comment 4: Language and grammatical revision

 Response:

 The entire manuscript has undergone a comprehensive language review to improve grammatical accuracy, sentence structure, clarity of ideas, and consistency of terminology across all sections.

 We greatly appreciate your thoughtful feedback, which has significantly improved the quality and clarity of the manuscript.

 Kind regards,

 Praewchit Ritthaisong

# Reviewer report: Reviewer response for version 1

**DOI:** 10.5256/f1000research.175507.r391377

**Published:** 2025-08-28

**Authors:** Puree Anantachoti

**Affiliations:** 1 Chulalongkorn University, Bangkok, Bangkok, Thailand

Dear Editor in Chief/ Corresponding Author, 

 I have reviewed your manuscript titled "Application of Value Stream Mapping to Design and Develop an Inventory Management System in a Hospital." Overall, this study presented an interesting and relevant application of Value Stream Mapping (VSM) within a hospital setting. The action research approach, which focuses on solving a practical problem and sharing the experience, is highly valuable for other healthcare institutions that might consider applying VSM to their own inventory management challenges. The potential for practical impact is a significant strength of this manuscript. However, I found that the manuscript needs to be improved not only in writing style and conciseness, English language proofreading, but also strengthen the research methodology part. Detailed comments are provided below.

**Abstract**

 1. Introduction in the abstract captured only one side of the problem.  As you mentioned “Insufficient drug reserves in hospitals ready for immediate use pose significant challenges in the management, procurement, and distribution of medications. Therefore, there is a critical need for improved resource management processes, ensuring a balanced and efficient medication reserve system. Lean Management is a systematic approach designed to eliminate waste, thereby reducing costs and enhancing efficiency, making it more effective than other management systems.”

 My issues are: 

 1.1 In the full manuscript, you are not only focused on insufficient, but also on over stockpiles too.

 1.2 The information presented is contradicted. You started with insufficient drug reserve, but your next supporting sentence talked about elimination waste.

 2. The objective was stated as “In this study, VSM was employed to comprehensively analyze the drug disbursement process within the medical supply warehouse managementsystem.”

 2.1 Spelling  managementsystem  change to management system

 2.2 The objective and results are not matched. The objective is to use the VSM as a framework to only analyzed the workflow process, but the result provide broader activities finally propose a solution.

 3. Shortening the introduction, but described more key methodology part e.g. who are involved participants, their qualification, key variables of interest, method of data collection (focus group, in-depth interview, consensus method, Delphi method???)

 4. Result part reflected that the key outcome is “work efficiency” which measuring by 1) number of steps in workflow and 2) type of work time reduction. This 2 key outcome were not at all mentioned in the introduction part.  Similarly, the introduction mentioned about shortage and over stockpiles, but the result did not include them.   

 5. Conclusion “Design of work systems by those involved. This is a tool that helps reduce waste and has been developed collaboratively to make operations run more smoothly.”

 5.1 This part should provide short summary to your objective.

 5.2 First sentence is not a sentence. Please correct.

**Introduction**

 6. Please shorten the introduction. When reviewing the literature, please summarize, compare and contrast them instead of narratively describing each of them.

 7. I would like to see you summarize key outcomes that align with the study's objectives stated in the introduction part.

 8. Sequence of story telling in the introduction part may have to shuffle. May be talking about problems and problem solving (from literature review) first, then problem specific to your hospital (which is the gap of the study), then objective.

 9. The objective stated in the last paragraph of the introduction is that“The objective of this study was to identify inefficiencies and challenges within the medical supply inventory management system, to reduce waste and optimize processes using Value Stream Mapping (VSM), and to design and develop an enhanced drug inventory management system to improve efficiency and effectiveness.”

 9.1 I think your ultimate goal is to develop new inventory management system. But the process to reach the goal is that you 1) have to analyzed current workflow to identify problems and 2) use VSM as a framework and involve stakeholders to propose new management system. Your first and second objectives are just processes to reach your ultimate objective.

**Methodology**

 10. I understand that to answer the objective, there were many steps of work. Please make sure that for each step, you provide clear methodology elements e.g.

 10.1 Study design Action research can be used as a study design as it’s a broad term. But for each specific step, you might use different design e.g. in-depth interview, focus group, consensus method etc. Please be more specific.

 10.2 Sample  

 - Qualification (eligibility criteria)

 -Number of samples (how many samples needed to ensure validity of answers)

 10.3 Measurement

 - What outcomes were you seeking in each step. How did you measure it. These outcomes should reflect what you showed in the result section. Also, you measured work time, whether you interview to get the time, or you retrieve time from computer system? If many product items generate their own time, how would you calculate the reported time?

 - Some outcome may need to be operationalized e.g. waste

 As a reader, I’m not sure whether waste refers to expired over stockpiles. Since I did not see you measuring waste in terms of volume or value of products, but you measuring work time. I am not sure if you refer wasting time as waste or not.

 - What question guide do you use

 - Whether the questions valid

 10.4 Data analysis

 What data analysis method were use?

**Results**

 11. The figure clearly portrait the workflow both current situation and proposed solution. But the color and the font size make it difficult to read.

 12. Reporting style

 Under the “Value stream mapping” topic.

 Though understand that the Value stream mapping compose of 3 parts, but part 1 also showed in part 3. Part 2 is relevant to Figure 1. When putting together as result, you should summarized things that can be combined to make it short but still keep its clarity.

 13.  After table 1, there is a word “working period” come out of nowhere. Please consider deleting it.

 14. Sub-topic Future work system

1. I suggested paraphrase “Future work system “ to “proposed work system”
2. Writing style. The result section is very long. You should synthesize first and laid down the content ที่ประมวลแล้ว in a clear and shorter version.
3. I’m not sure whether this proposed work system came from researchers’ opinions or from the participants’ consensus? Please clarify both in the methodology and in the result.

**Discussion**

 15. In general, 1) do not repeat results in discussion and 2) make it short for each issue raised.

 16. In the first discussion paragraph, please provide unique key findings or key strength of the study.

 17. Many previous studies showed in discussion should be described in the introduction. When mentioned about previous studies, their findings should be compared or contrasted with your findings.

 18. Limitation and suggestions (or recommendations) should be included under discussion section.

**Conclusion**

 The conclusion should be the last part of the manuscript’s content.

**Ethics and consent**

 You already mentioned about ethical approval at the very beginning of the methodology section. If this is the journal’s format, please delete the one in methodology section.  It should not be redundant.

Is the work clearly and accurately presented and does it cite the current literature?

Partly

If applicable, is the statistical analysis and its interpretation appropriate?

No

Are all the source data underlying the results available to ensure full reproducibility?

Partly

Is the study design appropriate and is the work technically sound?

Partly

Are the conclusions drawn adequately supported by the results?

Yes

Are sufficient details of methods and analysis provided to allow replication by others?

No

Reviewer Expertise:

Social pharmacy, Administrative pharmacy, Health and Pharmacy policy, Access to medicine, Regulatory Sciences

I confirm that I have read this submission and believe that I have an appropriate level of expertise to confirm that it is of an acceptable scientific standard, however I have significant reservations, as outlined above.

# Response

**Published:** 2025-09-30

**Authors:** Praewchit Ritthaisong

**Affiliations:** Khon Kaen University, Nai Mueang, Khon Kaen, Thailand

**Title:** **Author Response to Reviewers**

**Dear Reviewers**,

 We sincerely thank you for your careful reading, constructive comments, and valuable suggestions on our manuscript *“Application of value stream mapping to design and develop an inventory management system in a hospital.”* We have revised the manuscript accordingly. Below, we provide a point-by-point response.

**Abstract**

 1. Introduction in the abstract captured only one side of the problem.  As you mentioned “Insufficient drug reserves in hospitals ready for immediate use pose significant challenges in the management, procurement, and distribution of medications. Therefore, there is a critical need for improved resource management processes, ensuring a balanced and efficient medication reserve system. Lean Management is a systematic approach designed to eliminate waste, thereby reducing costs and enhancing efficiency, making it more effective than other management systems.”

 My issues are: 

 1.1 In the full manuscript, you are not only focused on insufficient, but also on over stockpiles too.

 1.2 The information presented is contradicted. You started with insufficient drug reserve, but your next supporting sentence talked about elimination waste.

**Response:** (Revised AbstractàIntroduction)

 1.1We thank the reviewer for this important observation. In the revised abstract, we have modified the introduction to mention both insufficient reserves and overstockpiling as dual challenges

 1.2We have rephrased the abstract to improve logical flow, presenting both shortage and overstock before linking to Lean Management and VSM

 2. The objective was stated as “In this study, VSM was employed to comprehensively analyze the drug disbursement process within the medical supply warehouse managementsystem.”

 2.1 Spelling  managementsystem  change to management system

 2.2 The objective and results are not matched. The objective is to use the VSM as a framework to only analyzed the workflow process, but the result provide broader activities finally propose a solution.

**Response: **(Revised Abstract)

 2.1 Corrected as suggested.

 2.2 We have revised the abstract objective to better align with the study’s scope and outcomes

 3. Shortening the introduction, but described more key methodology part e.g. who are involved participants, their qualification, key variables of interest, method of data collection (focus group, in-depth interview, consensus method, Delphi method???)

**Response:** (Revised Abstract -> method)

 As suggested, the abstract has been shortened, and key methodological details have been added. We briefly report the number and roles of participants, their qualifications, the main variables of interest, and data collection methods (focus groups discussion).

 4. Result part reflected that the key outcome is “work efficiency” which measuring by 1) number of steps in workflow and 2) type of work time reduction. This 2 key outcome were not at all mentioned in the introduction part.  Similarly, the introduction mentioned about shortage and over stockpiles, but the result did not include them.   

**Response:** (Revised Abstract -> Result)

 We appreciate this comment. The revised abstract now states that the study outcomes included workflow simplification (number of steps reduced) and time reduction. At the same time, references to shortages and overstock have been clarified as motivating problems, while results focus on the efficiency metrics that were directly measured.

 5. Conclusion “Design of work systems by those involved. This is a tool that helps reduce waste and has been developed collaboratively to make operations run more smoothly.”

 5.1 This part should provide short summary to your objective.

 5.2 First sentence is not a sentence. Please correct.

**Response:** (Revised Abstract -> conclusion)

 5.1 We revised the abstract conclusion to briefly restate the objective and demonstrate how it was achieved.

 5.2 Corrected to a complete sentence

**Introduction (**Revised Introduction)

 6. Please shorten the introduction. When reviewing the literature, please summarize, compare and contrast them instead of narratively describing each of them.

**Response: **We have shortened the introduction, synthesized related studies.

 7. I would like to see you summarize key outcomes that align with the study's objectives stated in the introduction part.

**Response:** We added a summary of the key outcomes in the introduction to ensure alignment between objectives and results.

 8. Sequence of story telling in the introduction part may have to shuffle. May be talking about problems and problem solving (from literature review) first, then problem specific to your hospital (which is the gap of the study), then objective.

**Response:** The introduction has been reorganized accordingly.

 9. The objective stated in the last paragraph of the introduction is that“The objective of this study was to identify inefficiencies and challenges within the medical supply inventory management system, to reduce waste and optimize processes using Value Stream Mapping (VSM), and to design and develop an enhanced drug inventory management system to improve efficiency and effectiveness.”

 9.1 I think your ultimate goal is to develop new inventory management system. But the process to reach the goal is that you 1) have to analyzed current workflow to identify problems and 2) use VSM as a framework and involve stakeholders to propose new management system. Your first and second objectives are just processes to reach your ultimate objective.

**Response: **We thank the reviewer for this clarification. The objective has been revised to emphasize the ultimate aim: “to design and develop a new medical supply inventory management system that is efficient and effective by analyzing current inventory management processes to identify inefficiencies, applying Value Stream Mapping (VSM) as a framework for designing the new system, and improving processes to reduce waste and enhance system efficiency.

**Methodology**

 10. I understand that to answer the objective, there were many steps of work. Please make sure that for each step, you provide clear methodology elements e.g.

 10.1 Study design Action research can be used as a study design as it’s a broad term. But for each specific step, you might use different design e.g. in-depth interview, focus group, consensus method etc. Please be more specific.

 10.2 Sample  

 - Qualification (eligibility criteria)

 -Number of samples (how many samples needed to ensure validity of answers)

 10.3 Measurement

 - What outcomes were you seeking in each step. How did you measure it. These outcomes should reflect what you showed in the result section. Also, you measured work time, whether you interview to get the time, or you retrieve time from computer system? If many product items generate their own time, how would you calculate the reported time?

 - Some outcome may need to be operationalized e.g. waste

 As a reader, I’m not sure whether waste refers to expired over stockpiles. Since I did not see you measuring waste in terms of volume or value of products, but you measuring work time. I am not sure if you refer wasting time as waste or not.

 - What question guide do you use

 - Whether the questions valid

 10.4 Data analysis

 What data analysis method were use?

**Response:**

**10.1 **We clarified that Action Research served as the overarching design. Specific methods included focus group discussions for problem identification, and consensus-building workshops for system design. **(**Revised methodology)

**10.2 **We added eligibility criteria and detailed the participant composition: pharmacists, pharmacy assistants, and warehouse staff (22 participants in total). **(**Revised Participants)

**10.3 **We clarified that process time was measured and validated against electronic system data. Waste was defined as non–value-added time. Expired stock was acknowledged but not measured; instead, waste in this study referred to workflow inefficiencies. The question guide used in focus groups was reviewed by three experts to ensure content validity. (Revised Procedures and Data Collection, Outcomes and Measurements)

**10.4 **We revised the methodology to specify descriptive statistics for time data, comparative analysis of workflow steps, and thematic content analysis for qualitative inputs **. (**Revised Data Analysis )

**Results**

 11. The figure clearly portrait the workflow both current situation and proposed solution. But the color and the font size make it difficult to read.

**Response:** Figure 1 was reformatted with larger fonts, clearer labels, for better readability.

 12. Reporting style

 Under the “Value stream mapping” topic.

 Though understand that the Value stream mapping compose of 3 parts, but part 1 also showed in part 3. Part 2 is relevant to Figure 1. When putting together as result, you should summarized things that can be combined to make it short but still keep its clarity.

**Response: **Reporting style under Value Stream Mapping is repetitive. Response: We have synthesized overlapping points, reduced redundancy, and reorganized subsections to maintain clarity and conciseness. **(**Revised Result )

 13.  After table 1, there is a word “working period” come out of nowhere. Please consider deleting it.

**Response:** Deleted as suggested.

 14. Sub-topic Future work system

 I suggested paraphrase “Future work system “ to “proposed work system”

 Writing style. The result section is very long. You should synthesize first and laid down the content ที่ประมวลแล้ว in a clear and shorter version.

 I’m not sure whether this proposed work system came from researchers’ opinions or from the participants’ consensus? Please clarify both in the methodology and in the result.

**Response:** Subheading has been changed to “Proposed work system.” We also clarified that the design was primarily researcher-developed, with input and validation from participants through focus group consensus. (Revised Proposed work system)

**Discussion** . (Revised Discussion)

 15. In general, 1) do not repeat results in discussion and 2) make it short for each issue raised.

**Response: **The discussion was revised to avoid repetition and concisely address each issue.

 16. In the first discussion paragraph, please provide unique key findings or key strength of the study.

**Response:** The first paragraph of the Discussion now highlights unique contributions

 17. Many previous studies showed in discussion should be described in the introduction. When mentioned about previous studies, their findings should be compared or contrasted with your findings.

**Response:** Relevant literature has been moved to the Introduction. In the Discussion, previous findings are explicitly compared and contrasted with our study’s outcomes.

 18. Limitation and suggestions (or recommendations) should be included under discussion section.

**Response:** We have merged the limitations and recommendations into the final part of the Discussion, highlighting potential resistance to change, IT requirements, and scalability.

**Conclusion**

 The conclusion should be the last part of the manuscript’s content.

**Response:** Corrected as suggested.

**Ethics and consent**

 You already mentioned about ethical approval at the very beginning of the methodology section. If this is the journal’s format, please delete the one in methodology section.  It should not be redundant.

**Response:** Revised as suggested. Ethical approval is now presented only in the Ethics section.

 We sincerely appreciate your time, effort, and valuable feedback, which have helped us improve the quality of our manuscript. We hope that the revised version meets your expectations, and we remain open to further suggestions.

 Sincerely,

 Praewchit and Nusaraporn

# Reviewer report: Reviewer response for version 1

**DOI:** 10.5256/f1000research.175507.r394960

**Published:** 2025-08-19

**Authors:** Rungpetch Sakulbumrungsil

**Affiliations:** 1 College of Pharmacy Administration, Thailand, Thailand; 2 Chulalongkorn University, Pathumwan, Thailand

This manuscript presents a valuable case study in the hospital inventory management. It offers a solid contribution to the field of lean operations improvement. The methodology is sound and the transformation plan is logically constructed. The outcomes, particularly the improvements in lead time, process time, and activity ratio, are well-documented.

**Areas for Revision**

**1. Introduction and Literature Review**

- The research problem and study objectives should be clearly stated at the beginning of the introduction. Currently, these are embedded within a long narrative, which may obscure the purpose of the study. Since the manuscript integrates the introduction and literature review, it is recommended to present the study objectives early, before detailing the literature.
- The literature review is lengthy and contains some repetition, particularly in the sections discussing international applications of VSM. To enhance clarity and conciseness, consider summarizing similar case studies in a comparative table or highlighting the key findings, settings, and outcomes.

- Clarify the novelty of your study compared to existing Thai studies. What distinguishes your approach, IT integration, Kanban system, etc.?

**2. Methods**

- Include a brief summary of participant demographics (e.g., number and roles of pharmacists, warehouse staff, assistants) to improve transparency and understanding of the context.
- Specify the data collection timeframe and duration of each research phase (e.g., planning, implementation, observation) more clearly to help readers understand the study period and process scope.

**4. Results**

- In Table 2, it would be helpful to clearly distinguish between processing time and waiting time for each step to better align with the result in the table 3.
- Consider replacing the labels “current” and “future” states with “before” and “after”, as the new system has already been implemented. This will more accurately reflect the temporal progression and outcomes of the intervention.

**5. Discussion**

- Expand on the sustainability and scalability of the improved system. How will the hospital ensure long-term adoption, staff training, regular data updates, and system evaluation? Address how the IT infrastructure and human resources will support ongoing use.
- Compare findings more clearly to previous Thai studies and global case studies, emphasizing what this study adds.

**6. Limitations**

- The limitations are thorough, but consider adding commentary on potential resistance to change in healthcare environments when implementing new IT systems.

**7. Conclusion**

- The conclusion is appropriate but consider emphasizing:
  - Practical policy implications (e.g., how this model can inform regional hospital networks).
  - The feasibility of replication in other small-to-mid-sized hospitals.
  - The importance of continued monitoring through Lean cycles (e.g., annual PDSA reviews).

Is the work clearly and accurately presented and does it cite the current literature?

Yes

If applicable, is the statistical analysis and its interpretation appropriate?

Yes

Are all the source data underlying the results available to ensure full reproducibility?

No source data required

Is the study design appropriate and is the work technically sound?

Yes

Are the conclusions drawn adequately supported by the results?

Yes

Are sufficient details of methods and analysis provided to allow replication by others?

Yes

Reviewer Expertise:

inventory management

I confirm that I have read this submission and believe that I have an appropriate level of expertise to confirm that it is of an acceptable scientific standard, however I have significant reservations, as outlined above.

# Response

**Published:** 2025-09-30

**Authors:** Praewchit Ritthaisong

**Affiliations:** Khon Kaen University, Nai Mueang, Khon Kaen, Thailand

**Title:** **Author Response to Reviewers**

**Dear Reviewers**,

 We sincerely thank you for your careful reading, constructive comments, and valuable suggestions on our manuscript *“Application of value stream mapping to design and develop an inventory management system in a hospital.”* We have revised the manuscript accordingly. Below, we provide a point-by-point response.

**1. Introduction and Literature Review**

- The research problem and study objectives should be clearly stated at the beginning of the introduction. Currently, these are embedded within a long narrative, which may obscure the purpose of the study. Since the manuscript integrates the introduction and literature review, it is recommended to present the study objectives early, before detailing the literature.
- The literature review is lengthy and contains some repetition, particularly in the sections discussing international applications of VSM. To enhance clarity and conciseness, consider summarizing similar case studies in a comparative table or highlighting the key findings, settings, and outcomes.
- Clarify the novelty of your study compared to existing Thai studies. What distinguishes your approach, IT integration, Kanban system, etc.?

**Response: **We appreciate this valuable suggestion. In the revised manuscript, we have restructured the introduction to state the research problem and study objectives explicitly at the beginning, ensuring that the study purpose is clearly presented before the literature review, highlighting the key findings, contexts, and outcomes for clarity and conciseness. We have also added integration of IT systems, the application of the Kanban method, and the comprehensive VSM-based redesign. **(**Revised **introduction)**

**2. Methods**

- Include a brief summary of participant demographics (e.g., number and roles of pharmacists, warehouse staff, assistants) to improve transparency and understanding of the context.
- Specify the data collection timeframe and duration of each research phase (e.g., planning, implementation, observation) more clearly to help readers understand the study period and process scope.

**Response:** We have added a concise description of participant demographics, including the number and roles of pharmacists, pharmacy assistants, and warehouse staff involved. In addition, the revised Methods section specifies the timeframe of data collection and provides details on the duration of each research (Revised **introduction** Method , Participants, VSM tool involves five steps,)

**4. Results**

- In Table 2, it would be helpful to clearly distinguish between processing time and waiting time for each step to better align with the result in the table 3.
- Consider replacing the labels “current” and “future” states with “before” and “after”, as the new system has already been implemented. This will more accurately reflect the temporal progression and outcomes of the intervention.

**Response:** Table 2 has been revised to clearly separate processing time and waiting time for each step, which aligns with the results presented in Table 3. Moreover, we have replaced the labels “current” and “future” states with “before” and “after,” to more accurately reflect the temporal progression and outcomes following the intervention.

**5. Discussion**

- Expand on the sustainability and scalability of the improved system. How will the hospital ensure long-term adoption, staff training, regular data updates, and system evaluation? Address how the IT infrastructure and human resources will support ongoing use.
- Compare findings more clearly to previous Thai studies and global case studies, emphasizing what this study adds.

**Response: **We have expanded the Discussion section to address sustainability and scalability. We also added a more explicit comparison between our findings, previous Thai studies, and global case studies, highlighting how our integrated e-Kanban–VSM approach with IT support provides a distinctive contribution **(**Revised **Dicussion, Sugestion** **)**

**6. Limitations**

- The limitations are thorough, but consider adding commentary on potential resistance to change in healthcare environments when implementing new IT systems.

**Response: **We have included additional commentary on the potential resistance to change in healthcare environments when implementing new IT-based systems. This provides a more balanced view of the challenges in applying our approach.( Revised Limitation)

**7. Conclusion**

- The conclusion is appropriate but consider emphasizing:
- Practical policy implications (e.g., how this model can inform regional hospital networks).
- The feasibility of replication in other small-to-mid-sized hospitals.
- The importance of continued monitoring through Lean cycles (e.g., annual PDSA reviews).

**Response:** We highlight the feasibility of replicating this system in other small- to mid-sized hospitals and stress the importance of ongoing monitoring using Lean cycles, such as annual PDSA reviews, to ensure continuous improvement. (Revised Suggestion)

 We sincerely appreciate your time, effort, and valuable feedback, which have helped us improve the quality of our manuscript. We hope that the revised version meets your expectations, and we remain open to further suggestions.

 Sincerely,

 Praewchit and Nusaraporn
